# The use of *Panax notoginseng* saponins injections after intravenous thrombolysis in acute ischemic stroke: a systematic review and meta-analysis

**DOI:** 10.3389/fphar.2024.1376025

**Published:** 2024-06-05

**Authors:** Yaoyuan Liu, Puyu Niu, Hongchang Ji, Zhe Chen, Jingbo Zhai, Xinyao Jin, Bo Pang, Wenke Zheng, Junhua Zhang, Fengwen Yang, Wentai Pang

**Affiliations:** ^1^ Evidence-Based Medicine Center, Tianjin University of Traditional Chinese Medicine, Tianjin, China; ^2^ First Teaching Hospital of Tianjin University of Traditional Chinese Medicine, National Clinical Research Center for Chinese Medicine Acupuncture and Moxibustion, Tianjin, China; ^3^ Haihe Laboratory of Modern Chinese Medicine, Tianjin University of Traditional Chinese Medicine, Tianjin, China; ^4^ School of Public Health, Tianjin University of Traditional Chinese Medicine, Tianjin, China

**Keywords:** *Panax notoginseng* saponins, intravenous thrombolysis, acute ischemic stroke, systematic review, meta-analysis

## Abstract

**Background:**

As a bioactive metabolite preparation widely used in acute ischemic stroke (AIS), the efficacy and safety of *Panax notoginseng* saponins injections (PNSI) in patients with AIS after intravenous thrombolysis remain to be evaluated.

**Methods:**

This study included randomized controlled trials published before 26 April 2024 in 8 databases. AIS patients who received intravenous thrombolysis were included. The control group receiving conventional treatment and the treatment group receiving additional PNSI. Primary outcomes were selected as mortality, disability, and adverse events. Secondary outcomes were selected as all-cause mortality, improvement of neurological deficit, quality of life, and cerebral injury indicators. The revised Cochrane Risk of Bias tool was used to assess risk of bias. Risk ratio (RR) and mean differences (MD) were calculated for binary variables and continuous variables, respectively, based on a 95% confidence interval (CI).

**Results:**

A total of 20 trials involving 1,856 participants were included. None of them reported mortality or disability. There was no significant difference in the adverse events [RR: 1.04; 95% CI: 0.60 to 1.81] and hemorrhagic transformation [RR: 0.99; 95% CI: 0.36 to 2.70] between the two groups. Compared to the control group, the treatment group had a better effect in neurological improvement assessed by National Institutes of Health Stroke Scale [MD: −2.91; 95% CI: −4.76 to −1.06], a better effect in activities of daily living changes in Barthel Index [MD: 9.37; 95% CI: 1.86 to 16.88], and a lower serum neuron-specific enolase level [MD: −2.08; 95% CI: −2.67 to −1.49].

**Conclusion:**

For AIS patients undergoing intravenous thrombolysis, the use of PNSI improved neurological deficits and enhanced activity of daily living in the short term without increasing the occurrence rate of adverse events. However, due to the moderate to very low certainty of evidence, it is advisable to conduct high-quality clinical trials to validate the findings of this study.

**Systematic Review Registration:**

https://www.crd.york.ac.uk/prospero/display_record.php?RecordID=466851, Identifier CRD42023466851

## 1 Introduction

Insufficient blood flow resulting from the occlusion of cerebral arteries leads to acute ischemic stroke (AIS), causing ischemic cerebral injury and tissue necrosis (Phipps and Cronin, 2020). The occurrence of AIS has resulted in increased death rates and functional limitations in the population, significantly impacting the wellbeing of individuals and imposing a substantial strain on the worldwide healthcare infrastructure (Ma et al., 2021; Tsao et al., 2023; Tu et al., 2023). At present, the main approaches for treating AIS involve reperfusion therapy and cerebral cytoprotection therapy, with the primary goals of reducing mortality and disability (Powers et al., 2019; Berge et al., 2021). Reperfusion therapy in AIS is primarily based on intravenous thrombolysis (IVT) (Peng et al., 2018; Powers et al., 2019; Berge et al., 2021), aiming to reopen blood vessels, restore blood supply, prevent ischemic cascades, and rescue neurovascular units in ischemic cerebral tissue (Moskowitz et al., 2010).

While IVT may be one of the most effective ways to provide relief for ischemia injury, ischemia-reperfusion (I/R) injury that occurs after vascular reperfusion may lead to secondary necrosis of cerebral tissue and adverse events represented by hemorrhagic transformation (Goncalves et al., 2022; Liu et al., 2023). Furthermore, incomplete tissue reperfusion caused by partial recanalization of cerebral arteries, as well as vessel reocclusion after IVT, affect the effectiveness of IVT (Tsivgoulis et al., 2023). Therefore, the development of cytoprotective agents directly targeting ischemic regions of cerebral tissue to alleviate potential I/R injury caused by vascular reperfusion has become a current research focus (Fisher and Savitz, 2022). Additionally, novel therapeutic strategies aimed at reducing the risk of vascular reocclusion after IVT are also emerging (Tsivgoulis et al., 2023).

*Panax notoginseng* (Burkill) F.H.Chen [Araliaceae; *Notoginseng radix et rhizoma*] is a medicinal plant widely used in China. Traditional Chinese medicine has found that it simultaneously possesses the functions of promoting blood circulation and stopping bleeding, and it is widely used in various conditions involving blood stasis and bleeding disorders (Shi et al., 2023; Wu et al., 2023). *Panax notoginseng* saponins (PNS) are the main bioactive metabolites of *P. notoginseng*, and their primary chemical metabolites include five types of dammarane-type saponins, namely, notoginsenoside R1, ginsenoside Rg1, ginsenoside Re, ginsenoside Rb1, and ginsenoside Rd (Sun et al., 2020). *In vivo* experiments indicate that PNS reduced the cerebral infarct volume in rat models of I/R injury, and improved neurological deficits. This effect may be associated with theirs ability to increase cerebral blood flow, alleviate excitotoxicity, decrease inflammatory responses and oxidative stress, and improve the permeability of the blood-brain barrier, demonstrating the therapeutic potential in AIS (Dong et al., 2017; Wang et al., 2017; 2018; Xu et al., 2018).

As the main clinical formulation of PNS, *P. notoginseng* saponins injections (PNSI, including Xuesaitong injection and Xueshuantong injection) have undergone several clinical trials and systematic reviews, indicating theirs ability to reduce the occurrence of disabilities and improve neurological deficits as well as activity of daily living (Dai et al., 2022; Shi et al., 2023). However, it is crucial to note that whether AIS patients have received or not received IVT has a significant difference on their prognosis and medication (National Institute of Neurological Disorders and Stroke rt-PA Stroke Study Group, 1995), and existing systematic reviews have not yet made a distinction among participants on this issue. Hence, the efficacy and safety of using PNSI in AIS patients undergoing IVT is still a question that clinicians and researchers urgently need answers to. This systematic review and meta-analysis aim to include randomized controlled trials to investigate this issue.

## 2 Materials and methods

The study protocol was registered with PROSPERO (International Prospective Register of Systematic Reviews), under the ID: CRD42023466851. The study adhered to the reporting principles outlined in the Preferred Reporting Items for Systematic Reviews and Meta-Analyses 2020 (PRISMA 2020) guidelines (Page et al., 2021).

### 2.1 Eligibility criteria

Inclusion criteria: 1) Study design: Randomized controlled trials were included. 2) Participants: Diagnosed with AIS and underwent IVT within 4.5 h. Supplementary Material S1 contains comprehensive diagnostic criteria of AIS. IVT must have been performed based on the guidelines (Peng et al., 2018; Berge et al., 2021). Participants of any age, gender, race, and region were included. 3) Intervention and control: The control group received the guideline-recommended conventional treatment for AIS, which included antiplatelet agents, anticoagulants, and statin drugs, in addition to IVT. PNSI was given to the treatment group along with conventional treatment. Supplementary Material S2 contains comprehensive details about PNSI. 4) Outcomes: Primary outcomes: Mortality and disability rates at the end of at least 3 months of follow-up, as well as the occurrence adverse events. Secondary outcomes: All-cause mortality during treatment, improvement of neurological deficit, quality of life, and cerebral injury-related indicators.

Exclusion criteria: 1) In addition to AIS, the participants also suffered from other neurological diseases, or severe diseases in systems other than the nervous system. 2) Pregnant or lactating women. 3) Participants receiving other traditional Chinese treatments, such as acupuncture and other botanical drugs. 4) Only one of the duplicate studies were retained. 5) Studies containing inaccurate or incomplete data, or those where a sufficient amount of data could not be extracted.

### 2.2 Information sources and search strategy

The research in both Chinese and English originated from the China National Knowledge Infrastructure Database (CNKI), VIP Database for Chinese Technical Periodicals (VIP), Wanfang Database (Wanfang), Chinese Biomedical Literature Database (SinoMed), Web of Science, MEDLINE, Embase, and Cochrane Library. Searches were conducted from the beginning of the databases to 26 April 2024. Combining subject terms with free-text terms was used for the search. Search queries are provided in Supplementary Material S3.

### 2.3 Selection process and data collection process

Two reviewers independently screened and extracted data from the literature. Discussions were held to resolve differences. The procedure involved initially eliminating duplicate studies, then examining the titles and abstracts of the studies, and excluding those that do not fulfill the criteria. Subsequently, conduct a comprehensive reading and screening of the remaining studies. The extraction of data involved obtaining the subsequent information: 1) Fundamental details of the research, encompassing the title, primary author, source, year and country of publication; 2) Study details, including patient information, intervention and control, dosage of medication, treatment duration, outcomes, and adverse events.

### 2.4 Study risk of bias assessment and certainty assessment

Using the revised Cochrane Risk of Bias tool (RoB 2) (Sterne et al., 2019), two reviewers individually evaluated the potential for the risk of bias in each study. Differences were settled by engaging in discussions with the third reviewer. To evaluate the certainty of evidence, a methodology known as GRADE was employed, classifying it as high, moderate, low, or very low (Guyatt et al., 2008). The certainty of evidence for each outcome was evaluated independently by two reviewers. A third reviewer was consulted to resolve differences. Supplementary Material S4 provides detailed rules for assessing the certainty of evidence.

### 2.5 Data analysis and synthesis

The study was analysed using Review Manager 5.4. Risk ratio (RR) was calculated for binary variables and mean differences (MD) was calculated for continuous variables, based on a 95% confidence interval (CI). The I^2^ statistic was used to evaluate heterogeneity, and a value of I^2^ greater than 50% indicated significant heterogeneity. In instances where there was no significant heterogeneity, the fixed-effects model was utilized for result pooling. Necessary subgroup analyses and sensitivity analyses were conducted for studies with significant heterogeneity. In case the underlying causes of heterogeneity could not be clarified, a random-effects model was employed to pool the findings. By excluding individual studies, sensitivity analyses assessed the robustness of the results. Funnel plots were utilized to evaluate publication bias when the number of studies surpassed 10. In addition, various PNSI dosages and treatment durations were analyzed in subgroups, and the difference between subgroups were compared.

## 3 Results

### 3.1 Selection and characteristics of studies

**FIGURE 1:**
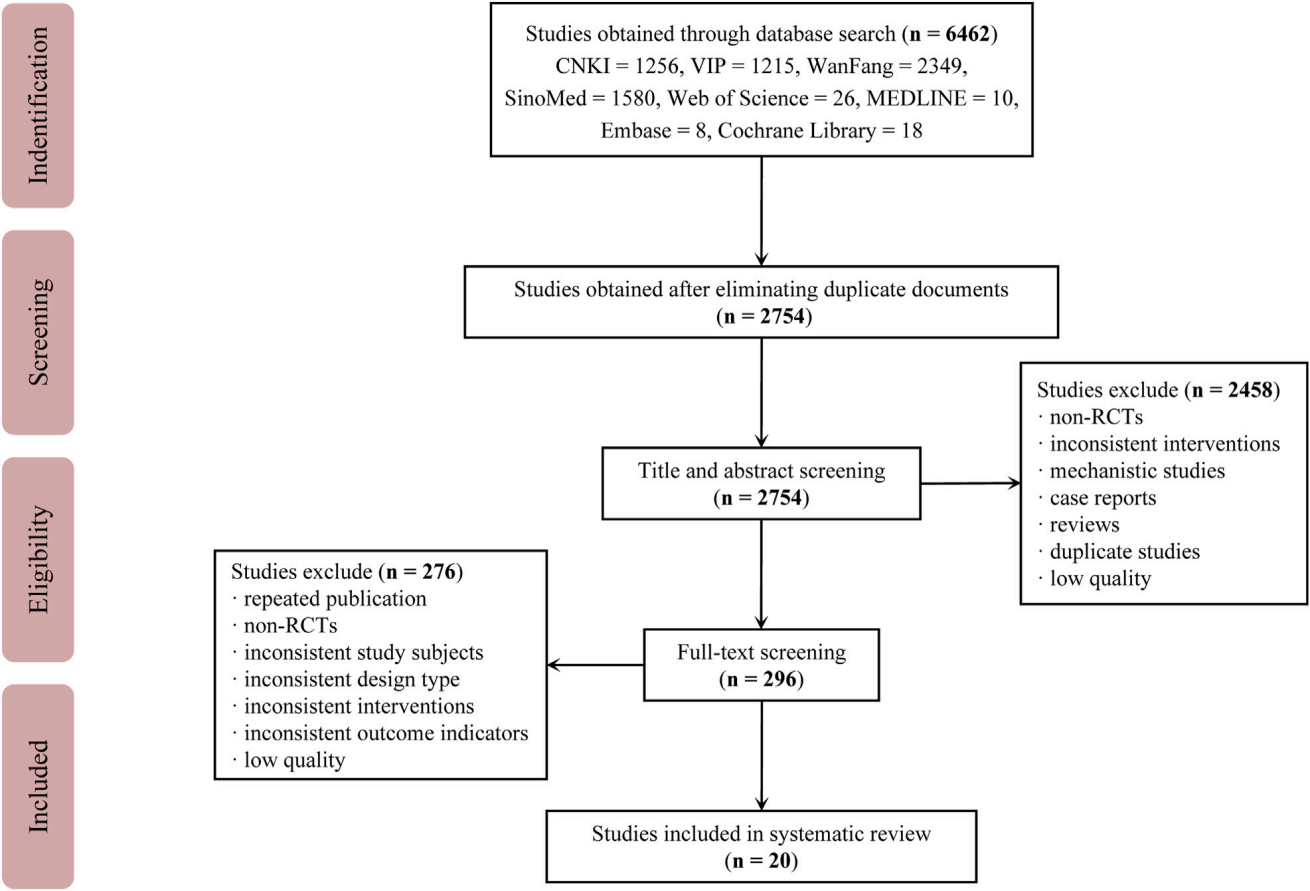
Study selection process Abbreviations: CNKI, China National Knowledge Infrastructure Database; VIP, VIP Database for Chinese Technical Periodicals; Wanfang, Wanfang Database; SinoMed, Chinese Biomedical Literature Database; RCTs, randomized controlled trials.

A total of 6,462 studies were retrieved from the databases. After removing duplicate studies, 2,754 studies remained. After examining the titles and abstracts, a total of 2,458 studies were eliminated, leaving behind 296 studies for thorough evaluation of full-texts. Out of these, 276 studies were subsequently excluded. Finally, 20 eligible studies were included. The study selection process is depicted in Figure 1. The studies included in this study were all conducted in China and reported in Chinese. A total of 1,916 participants were involved, with 978 in the treatment group and 938 in the control group (Chen, 2013; Xie, 2013; Chen and Chen, 2014; Cheng et al., 2014; Liu, 2015; Huo and Xie, 2018; Bian, 2019; Guan, 2019; Mo, 2019; Yan et al., 2020; Li, 2021; Liu and Zou, 2021; Liu et al., 2022; Luo et al., 2022; Zhang et al., 2022; Li and Fang, 2023; Zeng et al., 2023; Zhang, 2023; Li and Wang, 2024; Zhao et al., 2024). The average age of participants ranged from 56.5 to 69.17. In most trials, the number of males was higher than that of females. There were no significant differences found between the baselines described in any of the studies. Detailed information about the included studies is provided in Table 1.

**TABLE 1:** Information of the included studies.

Included studies	Sample size		Sex (M/F)		Age		Onset time (h)		Intervention		Course of treatment (d)	Outcomes
					(Mean ± standard deviation)							
	T	C	T	C	T	C	T	C	T	C		
Bian (2019)	35	35	20/15	21/14	—	—	≤4.5	≤4.5	PNSI 350 mg + CT	CT	15	④
Chen (2013)	80	70	57/23	45/25	62.8 ± 5.3	63.4 ± 4.9	≤4.5	≤4.5	PNSI 420 mg + CT	CT	3	②
Chen and Chen (2014)	80	80	51/29	48/32	63.89 ± 8.76	64.13 ± 9.02	≤4.5	≤4.5	PNSI 250 mg + CT	CT	14	②
Cheng et al. (2014)	33	30	18/15	17/13	59.8 ± 6.7	60.6 ± 6.4	≤4.5	≤4.5	PNSI 400 mg + CT	CT	14	②
Guan (2019)	39	39	21/18	17/22	69.17 ± 3.36	68.92 ± 2.41	3.37 ± 1.09	3.42 ± 1.17	PNSI 200 mg + CT	CT	15	②
Huo and Xie (2018)	60	30	36/24	18/22	56.5 ± 0.3	57.1 ± 0.2	≤4.5	≤4.5	PNSI 300 mg + CT	CT	42	①
Li (2021)	45	45	26/19	25/20	62.1 ± 11.7	61.3 ± 12.6	3.8 ± 0.9	3.6 ± 1.1	PNSI 140 mg + CT	CT	14	②③④
Li and Fang (2023)	43	43	25/18	24/19	56.58 ± 4.37	57.05 ± 4.43	1.24 ± 0.35	1.28 ± 0.37	PNSI 200 mg + CT	CT	10	①③
Li and Wang (2024)	50	50	27/23	26/24	61.54 ± 12.62	62.06 ± 12.73	3.42 ± 0.95	3.39 ± 0.91	PNSI 70 mg + CT	CT	14	④
Liu (2015)	40	40	25/15	28/12	—	—	≤4.5	≤4.5	PNSI 500 mg + CT	CT	28	①
Liu and Zou (2021)	45	45	25/20	28/17	58.46 ± 4.96	58.37 ± 4.84	≤4.5	≤4.5	PNSI 400 mg + CT	CT	14	①②
Liu et al. (2022)	40	44	23/17	25/19	57.19 ± 5.88	57.10 ± 5.81	1.09 ± 0.38	1.19 ± 0.40	PNSI 200 mg + CT	CT	10	②③
Luo et al. (2022)	30	30	15/15	16/14	64.54 ± 6.05	64.60 ± 6.03	2.02 ± 0.21	1.96 ± 0.20	PNSI 500 mg + CT	CT	14	①④
Mo (2019)	35	35	20/15	19/16	56.5 ± 3.8	56.2 ± 3.7	2.2 ± 0.3	2.1 ± 0.5	PNSI 450 mg + CT	CT	10	②
Xie (2013)	30	30	17/13	15/15	61.5 ± 10.2	62.3 ± 13.1	≤4.5	≤4.5	PNSI 200 mg + CT	CT	14	③
Yan et al. (2020)	46	45	30/16	29/16	63.17 ± 3.45	62.57 ± 3.24	≤4.5	≤4.5	PNSI 300 mg + CT	CT	14	①②③④
Zeng et al. (2023)	45	45	23/22	25/20	66.32 ± 5.73	66.95 ± 5.45	3.37 ± 0.27	3.32 ± 0.31	PNSI 70 mg + CT	CT	14	②③
Zhao et al. (2024)	100	100	59/41	56/44	64.66 ± 7.48	64.63 ± 6.22	≤4.0	≤4.0	PNSI 300 mg + CT	CT	28	①
Zhang et al. (2022)	60	60	40/20	38/22	61.9 ± 7.7	61.2 ± 7.4	≤3.0	≤3.0	PNSI 400 mg + CT	CT	7	②③
Zhang (2023)	42	42	18/24	23/19	61.45 ± 4.15	62.47 ± 4.31	2.62 ± 0.48	2.75 ± 0.41	PNSI 300 mg + CT	CT	14	②④

Abbreviations: T, treatment group; C, control group; M, male; F, female; h, hour; PNSI, *panax notoginseng* saponins injections; CT, conventional treatment; d, day; ① adverse events; ② neurological improvement changes in National Institutes of Health Stroke Scale; ③ activities of daily living changes in Barthel Index; ④ serum neuron-specific enolase level.

### 3.2 Risk of bias in studies

**FIGURE 2:**
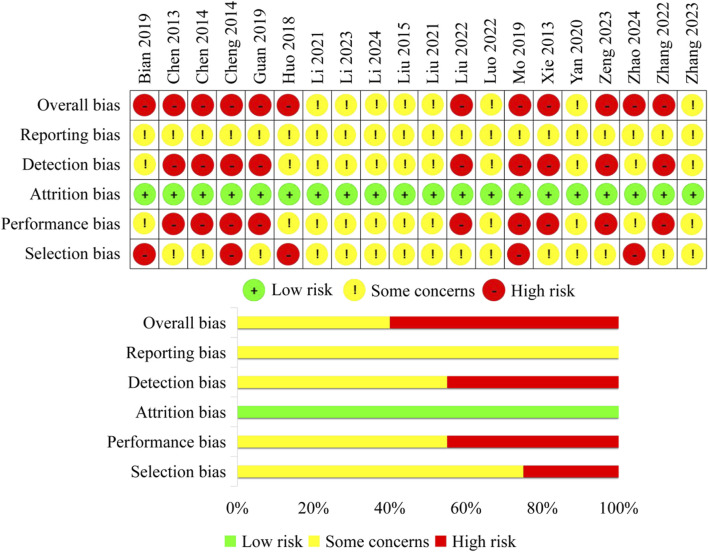
Risk of bias of included studies.

1) In terms of selection bias, fifteen studies that did not clearly indicate whether allocation concealment was performed and had evenly distributed baseline characteristics among groups were rated as some concerns. Five studies that did not conduct allocation concealment were rated as high risk. 2) None of the studies used any blinding methods. Therefore, nine studies that only reported subjective outcomes were rated as high risk in terms of performance bias, while the other studies were rated as some concerns. 3) All the included studies had complete data with no missing information, therefore, the attrition bias was rated as low risk for all of them. 4) For subjective outcomes, if the assessor is aware of the intervention, it may potentially influence the measurement of the outcomes. Therefore, nine studies that only reported subjective outcomes were rated as high risk in terms of detection bias, while the other studies were rated as some concerns. 5) In the included studies, all the expected outcomes were reported. However, due to the lack of pre-published research protocols, the reporting bias of all studies was rated as some concerns. Finally, the overall bias for eight studies was rated as some concerns, while twelve studies were rated as high risk. Finally, the overall bias for eight studies was rated as some concerns, while twelve studies were rated as high risk (Figure 2).

### 3.3 Primary outcomes

#### 3.3.1 Mortality and disability rates at the end of follow-up

None of the trials reported mortality or disability at the end of follow-up.

#### 3.3.2 Adverse events

**FIGURE 3:**
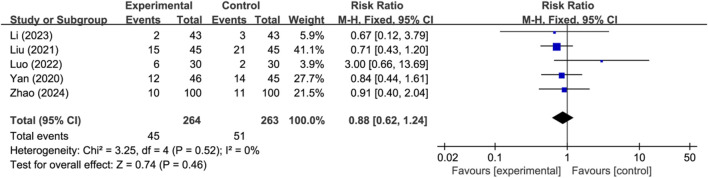
Meta-analysis results of adverse events.

Seven studies reported the occurrence of adverse events, with two studies reporting no adverse events and three studies reporting specific adverse events (*n* = 527). Analysis using a fixed-effects model indicated that there was no significant difference in the occurrence of adverse events between the two groups (RR: 0.88; 95% CI: 0.62 to 1.24; *p* = 0.46, I^2^ = 0%) (Figure 3).

**FIGURE 4:**
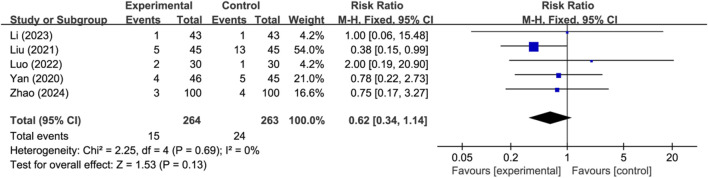
Meta-analysis results of hemorrhagic transformation.

Five studies reported the occurrence of hemorrhagic transformation (*n* = 527). Analysis using a fixed-effects model indicated there was no significant difference in the occurrence of hemorrhagic transformation between the two groups (RR: 0.62; 95% CI: 0.34 to 1.14; *p* = 0.13, I^2^ = 0%) (Figure 4). Other adverse events were mild and self-limiting, mainly including allergy, gastrointestinal reaction, and dizziness.

### 3.4 Secondary outcomes

#### 3.4.1 All-cause mortality during treatment

None of the trials reported all-cause mortality during treatment.

#### 3.4.2 Improvement of neurological deficit

**FIGURE 5:**
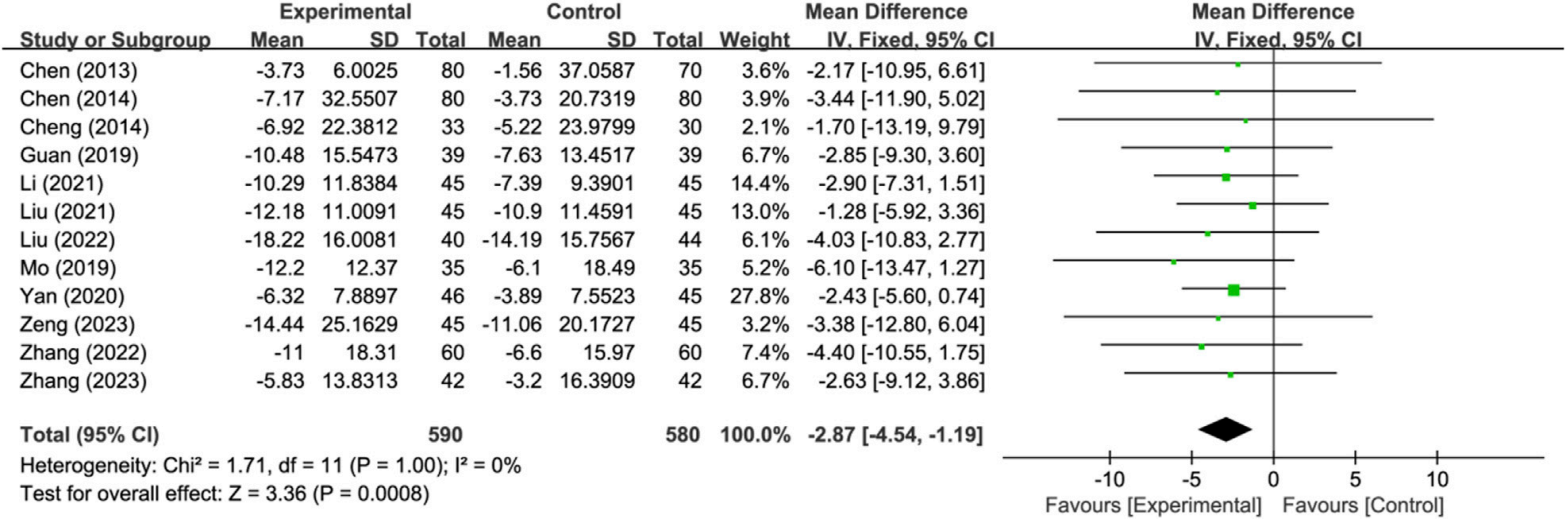
Meta-analysis results of neurological improvement changes in National Institutes of Health Stroke Scale.

Twelve studies reported neurological improvement changes in National Institutes of Health Stroke Scale (NIHSS) (*n* = 1170). Analysis using a fixed-effects model indicated that compared to the control group, the treatment group had a better effect in terms of neurological improvement changes in NIHSS, and the statistical difference was significant (MD: −2.87; 95% CI: −4.54 to −1.19; *p* = 0.0008, I^2^ = 0%) (Figure 5).

#### 3.4.3 Activities of daily living changes in Barthel Index

**FIGURE 6:**
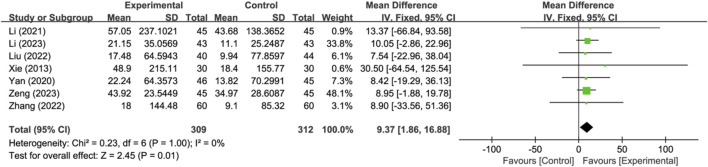
Meta-analysis results of activities of daily living changes in Barthel Index.

Seven studies reported activities of daily living changes in Barthel Index (*n* = 621). Analysis using a fixed-effects model indicated that compared to the control group, the treatment group had a better effect in activities of daily living changes in Barthel Index, and the statistical difference was significant (MD: 9.37; 95% CI: 1.86 to 16.88; *p* = 0.01, I^2^ = 0%) (Figure 6).

#### 3.4.4 Cerebral injury-related indicator

**FIGURE 7:**
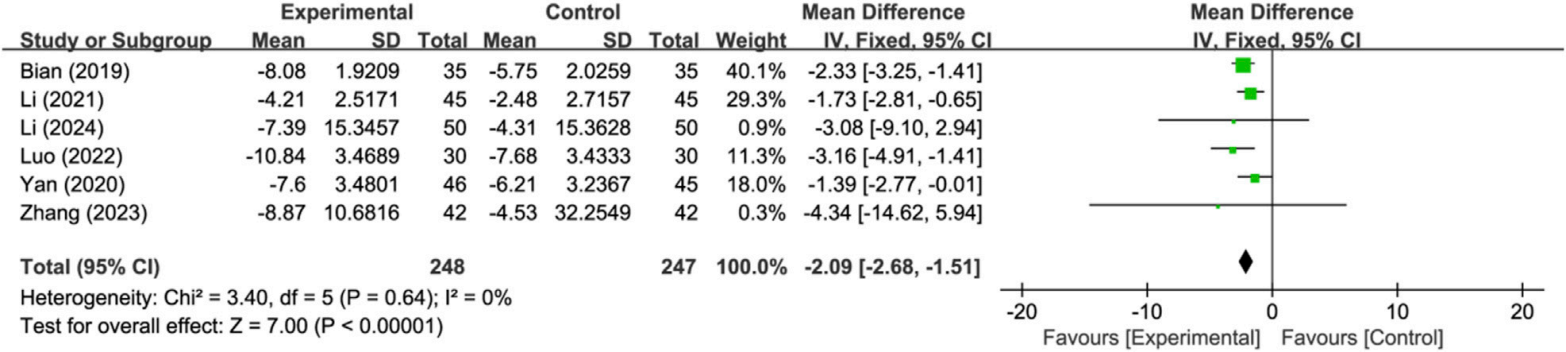
Meta-analysis results of serum neuron-specific enolase level.

Six studies reported serum neuron-specific enolase level as a cerebral injury-related indicator (*n* = 495). Analysis using a fixed-effects model indicated that compared to the control group, the treatment group had a lower serum neuron-specific enolase level, and the difference between the two was significant (MD: −2.09; 95% CI: −2.68 to −1.51; *p* < 0.00001, I^2^ = 0%) (Figure 7).

### 3.5 Subgroup analysis and sensitivity analysis

**FIGURE 8:**
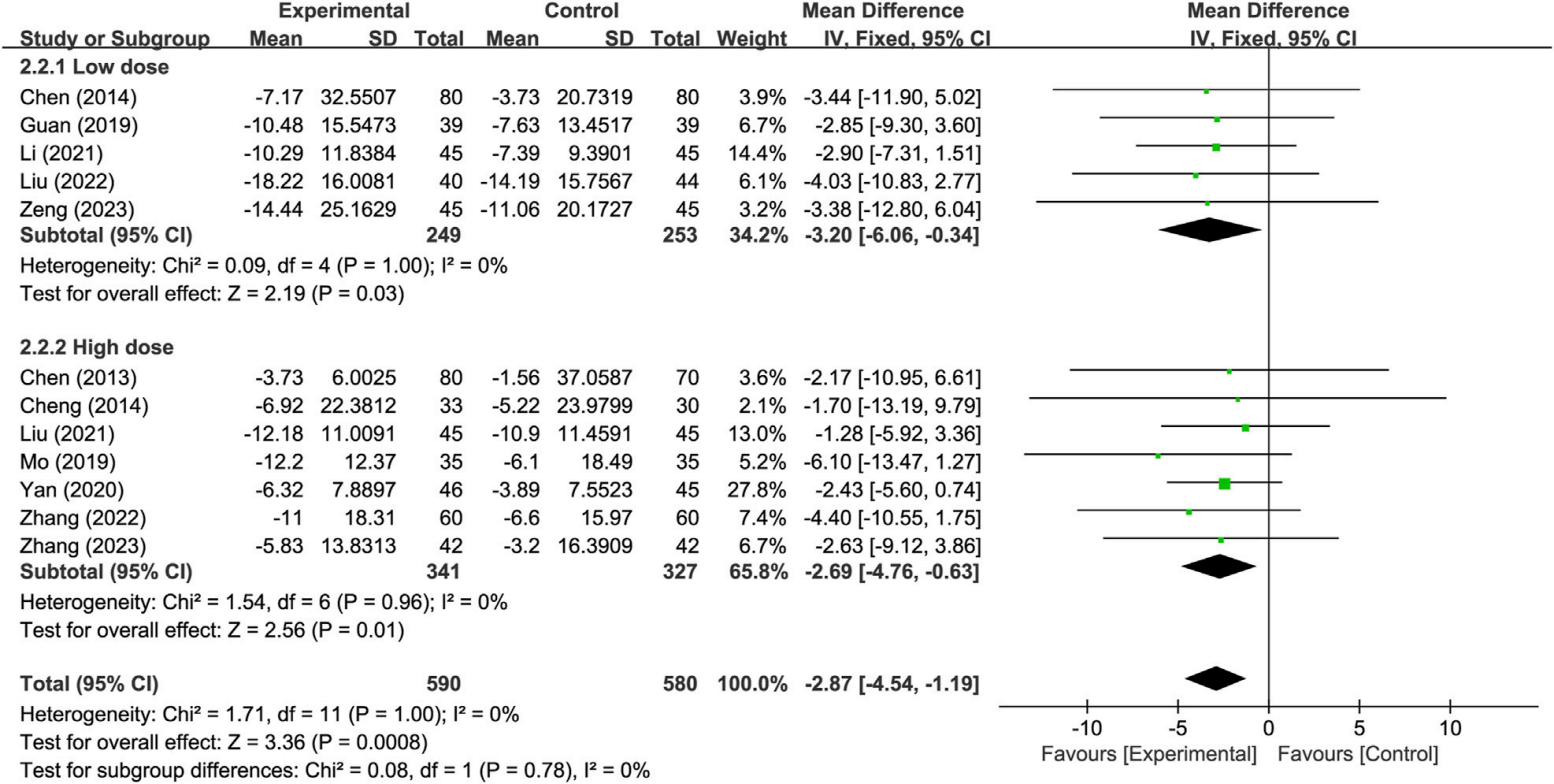
Subgroup analysis based on different PNSI dosages.

Subgroup analysis was conducted based on different PNSI dosages, with a daily dose of 300 mg or less classified as the low-dose subgroup and a daily dose exceeding 300 mg classified as the high-dose subgroup. The results indicate that, for the neurological improvement changes in NIHSS, the treatment group in each subgroup outperformed the control group, and showing significant difference. There were no significant difference observed between subgroups (*p* = 0.78) (Figure 8).

**FIGURE 9:**
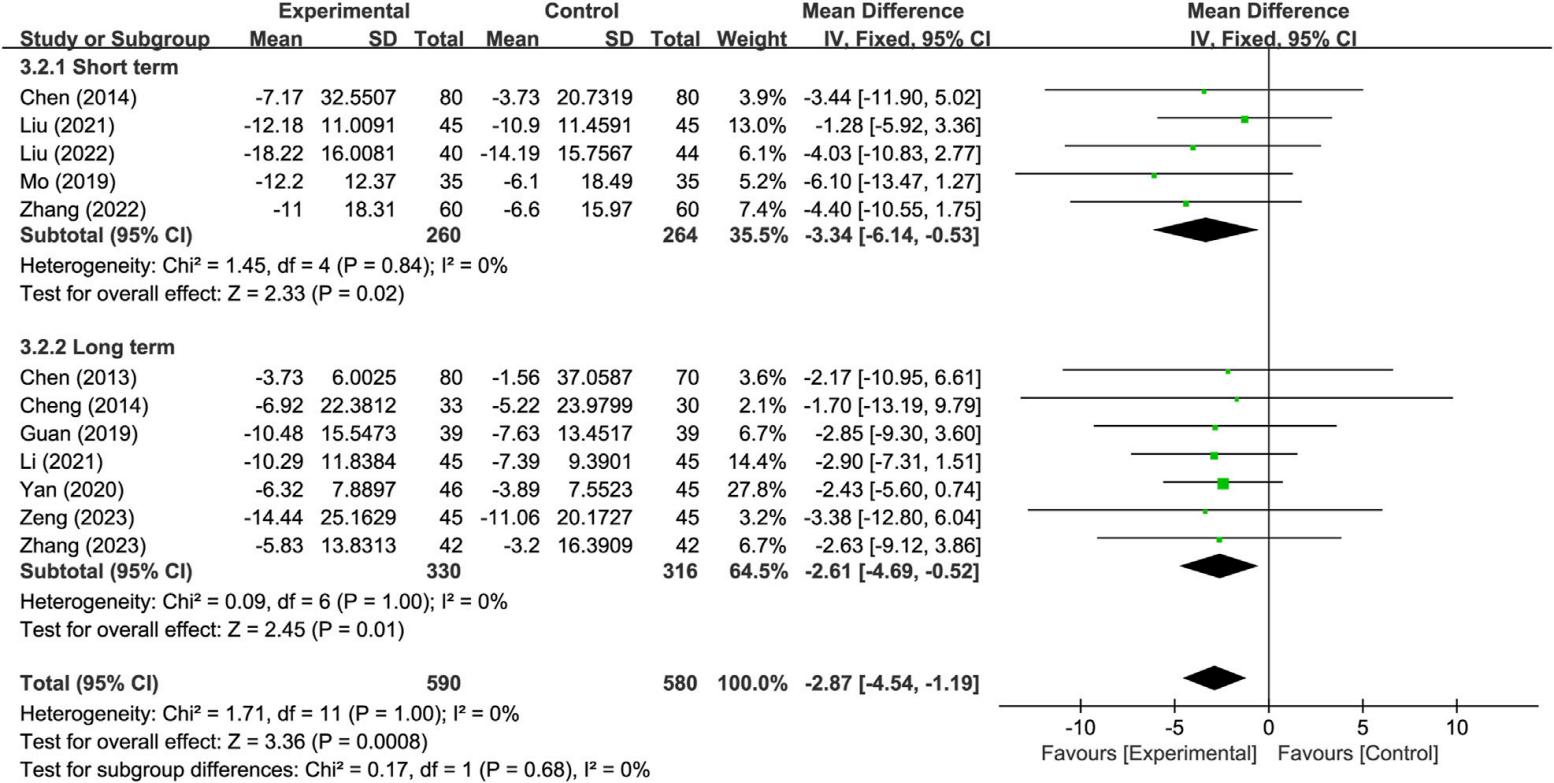
Subgroup analysis based on different treatment durations.

Another subgroup analysis was conducted based on different treatment durations, with 14 days or less classified as the short-term treatment subgroup and more than 14 days classified as the long-term treatment subgroup. The results indicate that, for the neurological improvement changes in NIHSS, the treatment group in each subgroup outperformed the control group, and showing significant difference. There were no significant difference observed between subgroups (*p* = 0.68) (Figure 9).

The sensitivity analyses showed that the meta-analysis results were not affected by removing any individual study, indicating the robustness of the results.

### 3.6 Publication bias

**FIGURE 10:**
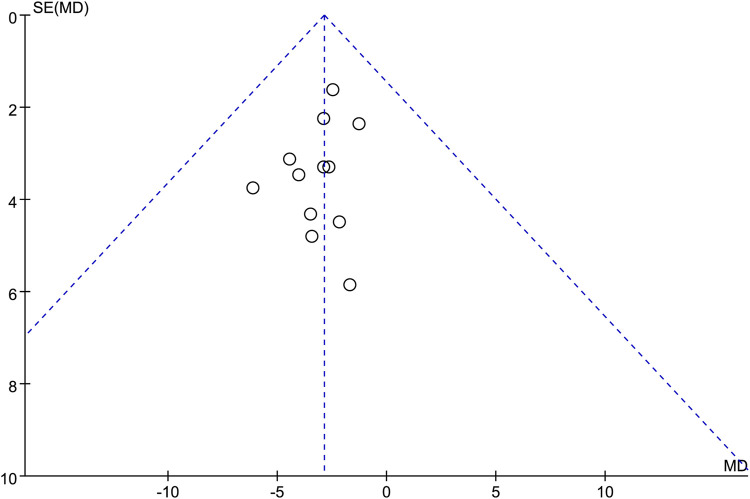
Funnel plots of the neurological improvement changes in National Institutes of Health Stroke Scale.

Funnel plot was used to assess the potential publication bias in the neurological improvement changes in NIHSS. The funnel plot was generally symmetrical, suggesting that there may be no publication bias in this outcome (Figure 10).

### 3.7 Certainty of evidence

Table 2 displays the assessment of evidence certainty. According to the results, the evidences for neurological improvement changes in NIHSS and activities of daily living changes in Barthel Index were regarded as moderate certainty; the occurrence of adverse events, and serum neuron-specific enolase level were regarded as low certainty; the occurrence of hemorrhagic transformation was regarded as very low certainty. Based on the included studies, the evidences for the primary outcome of mortality and disability rates at the end of follow-up, as well as the secondary outcome of all-cause mortality during treatment were not available.

**TABLE 2:** Certainty of evidence.

Outcomes	Certainty assessment						No. Of participants		Effect		Certainty
	No. S	Study design	Inconsistency	Indirectness	Imprecision	Publication bias	Treatment group	Control group	Relative (95% CI)	Absolute (95% CI)	
Occurrence of adverse events	7	serious^a^	not serious	not serious	serious^b^	N/A	264	263	RR: 0.88, 0.62 to 1.24	N/A	⊕⊕○○ Low
Occurrence of hemorrhagic transformation	5	serious^a^	not serious	not serious	serious^b^	N/A	264	263	RR: 0.62, 0.34 to 1.14	N/A	⊕○○○ Very low
Neurological improvement changes in NIHSS	12	serious^a^	not serious	not serious	not serious	not serious	590	580	N/A	MD: -2.87, −4.54 to −1.19	⊕⊕⊕○ Moderate
Activities of daily living changes in Barthel Index	7	serious^a^	not serious	not serious	not serious	N/A	309	312	N/A	MD: 9.37, 1.86 to 16.88	⊕⊕⊕○ Moderate
Serum neuron-specific enolase	6	serious^a^	not serious	not serious	serious	N/A	248	247	N/A	MD: -2.09, −2.86 to −1.51	⊕⊕○○ Low

Abbreviations: No. S: number of studies; CI: confidence interval; RR: risk ratio; MD: mean difference.

^a^ Two-thirds of the information came from some concerns.

^b^ The 95% CI, crossed the null line.

## 4 Discussion

### 4.1 Interpretation of the results

This study evaluated the efficacy and safety of using PNSI in patients with AIS after IVT. A total of 20 trials involving 1,916 participants were included. The safety of traditional Chinese medicine injections has always been in the spotlight. Regarding the safety of PNSI, the results of meta-analysis showed that there was no statistically significant difference between the treatment group and the control group in terms of adverse events and hemorrhagic transformation (low and very low certainty of evidence). Taking into account the data from post-market safety monitoring (Li et al., 2018; XinYao et al., 2020), it can be conservatively stated that PNSI is unlikely to increase the incidence of adverse events in AIS patients receiving IVT treatment. More clinical trials and safety monitoring are needed to provide robust evidence for the safety of PNSI.

In terms of the efficacy of PNSI, this study aimed to evaluate the efficacy from various aspects, including mortality, disability, improvement in neurological deficits, daily life activities, and cerebral lesion. However, based on the available data, it is not yet possible to assess the effect of PNSI on mortality and disability in AIS patients undergoing IVT. Results of the meta-analysis indicate that after the treatment duration, PNSI were significantly associated with greater improvement in neurological deficits (moderate evidence certainty), and better performance in activities of daily living (moderate evidence certainty) in AIS patients undergoing IVT. These results suggest that the addition of PNSI after IVT significantly associated with better improvement in the overall neurological deficits and activities of daily living for the short term. In addition, compared with the control group, the treatment group had a lower serum neuron-specific enolase level, with a significant statistical difference (low certainty of evidence). It can be seen that the use of PNSI in AIS patients after IVT may be associated with milder cerebral lesion.

Subgroup analysis of different PNSI doses was conducted in this study. Compared to the control group, both the low-dose subgroup and the high-dose subgroup of PNSI significantly improved neurological deficits, with no significant difference between the two subgroups. Subgroup analysis based on different treatment duration was also conducted in this study. Compared to the control group, both the short-term subgroup and the long-term subgroup of PNSI significantly improved neurological deficits, with no significant difference between the two subgroups. The above results suggest that a PNSI treatment duration lasting more than 14 days may not provide additional benefits to patients in terms of improving neurological deficits. The use of high-dose PNSI may not necessarily bring additional benefits to improving neurological deficits, and a daily dose of 300 mg or less of PNSI may already be sufficient. It should be noted that since the evaluation of neurological deficits was conducted at the end of the treatment, different evaluation times may also impact the results. Therefore, head-to-head controlled trials of PNSI should be conducted to further clarify the potential impact of dose and duration on efficacy.

### 4.2 Quality of evidence

In order to provide references for clinical practice, this study conducted a detailed assessment of the quality of evidence for outcomes. Firstly, an assessment of the risk of bias in the included studies was conducted. It was found that more than two-thirds of the overall bias risks for all outcomes were rated as some concerns. As a result, the certainties for all outcomes were downgraded by one level. Secondly, inconsistency of evidence was assessed based on heterogeneity evaluation. Since all outcomes showed no significant heterogeneity, the certainties of all outcomes were not downgraded due to inconsistency issues. Thirdly, there were no significant differences in characteristics of the included studies. Therefore, the certainties of all outcomes were not downgraded due to issues of indirectness. Fourthly, in terms of imprecision, if the 95% CI for the outcomes crossed the null line, or if the sample size for the outcome was less than the optimal information size (400), the certainty assessment was downgraded by one level. Therefore, the certainties of two outcomes were downgraded by one level. Fifthly, no significant publication bias was found, and there was no certainty of the outcomes being downgraded due to publication bias. Finally, the certainty assessment of the two outcomes with a total number of studies less than seven was downgraded by one level.

### 4.3 Implications of the results

Although PNSI has been used in AIS patients for many years, there has been a persistent lack of systematic review for the clinical use of PNSI in AIS patients receiving IVT. Its efficacy and safety remain to be futher evaluated. According to the results of this study, PNSI significantly improved neurological deficits and enhanced activities of daily living in the short term without increasing the occurrence rate of adverse events and hemorrhagic transformations. For AIS patients receiving IVT, the addition of PNSI to conventional treatment may bring additional benefits in the short term. Although the qualities of evidence were only rated as moderate to very low, this study still provides physicians with some reference evidence for the use of PNSI and lays the foundation for further in-depth research.

The potential pharmacological effects of PNSI in AIS patients receiving IVT may include: 1) reducing the risk of hemorrhagic transformation after IVT by improving the permeability of the blood-brain barrier (Liu et al., 2021); 2) mitigating inflammation in the cerebral ischemic lesion by inhibiting the microglia activation and the release of pro-inflammatory mediators mediated by microglia/macrophages and reducing leukocyte adhesion (Zhang et al., 2016; Shi et al., 2017; Wang et al., 2017; Xu et al., 2018); 3) reducing glutamate level, enhancing the expression of glutamate transporter 1 in neuroglial cells, and mitigating excitotoxicity of neurons (Wang et al., 2017; 2018); 4) inhibiting free radical generation, promoting the synthesis of antioxidant enzymes, and alleviating oxidative stress (Zhou et al., 2014; Dong et al., 2017). PNS may inhibit the activation of the NLRP3 inflammasome through the PINK1/Parkin pathway, promote mitophagy, and alleviate cerebral I/R injury in rats (Xiao et al., 2022). Furthermore, PNS may activate the Nrf2 antioxidant signaling pathway through the PI3K/Akt pathway and prevent the disruption of the blood-brain barrier induced by oxygen-glucose deprivation/reperfusion *in vitro* (Hu et al., 2018). Proteomics and transcriptomics have revealed seven key transcription factors, including ERCC2 and NR4A3, which mediate the protection of PNS against cerebral I/R injury. These factors may play a crucial role in PNS alleviating I/R injury (Zhang et al., 2021). As a potential multi-target drug for the treatment of AIS, PNS hold broad research prospects.

### 4.4 Strengths and limitations

This study started from urgently needing to address clinical issues and focused on the use of PNSI in AIS patients after IVT. Through systematic review and meta-analysis, it is pointed out that based on existing evidence, PNSI may bring short-term benefits in improving neurological deficits and activity of daily living for AIS patients receiving IVT without increasing the incidence of adverse events. In addition, this study also preliminarily explored possible dose-response and duration-response relationships.

This study also has some limitations. Due to design defects, a limited number of studies and small sample sizes in the included trials, the certainties of evidence were rated only from moderate to very low. This lowers the credibility of the evidence, and existing conclusions need to be approached with caution. Due to the lack of long-term follow-up for participants in existing studies, this study could not evaluate the long-term efficacy and safety of PNSI. Moreover, due to PNSI being approved for the treatment of AIS only in China, all included trials were conducted in China, and no studies from other countries were included. This reduces the applicability of the evidence to other countries or regions.

Some issues identified during the review process may also serve as references for future trial designers. The timing of PNSI administration may have an impact on the efficacy and safety of AIS patients undergoing IVT, but none of the included studies reported on this aspect. For ischemic stroke, the location and size of cerebral infarction greatly affect clinical manifestations and prognosis. Although some studies have reported these aspects, they have not specifically analyzed the different possible effects on outcomes, which leads to potential clinical heterogeneity. Furthermore, NIHSS is the most commonly used scale for clinical evaluation of neurological function in AIS, and most of the included studies use it to assess neurological deficits. Nevertheless, this scale can only reflect the overall neurological deficit, and it is difficult to reflect specific neurological deficits such as limb motor function, cognitive function, and language function, which reduces the specificity and clinical applicability of the results.

### 4.5 Future perspectives

The lack of high-quality clinical trials is a common issue in the field of ethnopharmacology research. Randomized controlled trials with large samples and long-term follow-up that are rigorously designed and implemented often require higher financial and time costs, which may be the main reason for the lack of high-quality trial in included studies. In order to enhance the certainty of evidence and the generalizability of the findings, it is recommended that relevant pharmaceutical companies and research institutions increase their investment in research and conduct rigorously designed multi-center, large-sample, double-blind randomized controlled trials in different countries to evaluate the efficacy and safety of PNSI in AIS patients undergoing IVT. In trial design, it is recommended to include outcomes reflecting long-term efficacy, such as mortality and disability rates. It is also recommended to adopt specific examination methods to reflect specific manifestations of neurological function. Future studies should record the specific time of PNSI administration in detail. It is important not only to report the location and size of cerebral infarction but also to analyze their specific impact on efficacy, avoiding clinical heterogeneity. Considering the role of PNSI in alleviating cerebral ischemic and I/R injury (Zhang et al., 2016; Shi et al., 2017; Wang et al., 2017; 2018; Xu et al., 2018), the mechanisms of PNSI in AIS patients undergoing IVT require further investigation.

## 5 Conclusion

For the AIS patients undergoing IVT, PNSI significantly improved neurological deficits and enhanced activities of daily living in the short term without increasing the occurrence rate of adverse events and hemorrhagic transformations. Due to the moderate to very low certainty of the evidence, it is imperative to conduct high-quality and large-sample sizes trials to validate the findings of this study.

## Data Availability

The original contributions presented in the study are included in the article/Supplementary Material, further inquiries can be directed to the corresponding authors.

## Abbreviations

AIS, acute ischemic stroke; CI, confidence interval; I/R, ischemia-reperfusion; IVT, intravenous thrombolysis; NIHSS, National Institutes of Health Stroke Scale; PNS, *Panax notoginseng* saponins; PNSI, *Panax notoginseng* saponins injections; RR, risk ratio; MD, mean differences.

## References

[B1] Berge E. Whiteley W. Audebert H. De Marchis G. M. Fonseca A. C. Padiglioni C. (2021). European stroke organisation (eso) guidelines on intravenous thrombolysis for acute ischaemic stroke. Eur. Stroke J. 6, 1–62. 10.1177/2396987321989865 PMC7995316 33817340

[B2] Bian H. (2019). The impact and mechanism study of Xueshuantong combined with alteplase treatment on the neurological function of patients with acute ischemic stroke. J. Health must-Read 221.

[B3] Chen Y. (2013). Xueshuantong clinical observation on the treatment of the acute phase of ischemic stroke. Asian Pac Trad. Med. 157–158. 10.3969/j.issn.1673-2197.2013.05.082

[B4] Chen Z. Chen H. (2014). Analysis of influential factors on the efficacy of Xuesaitong injection in treating patients with ischemic stroke. J. Hainan Med., 2575–2577. 10.3969/j.issn.1003-6350.2014.17.1005

[B5] Cheng M. Li H. Qi S. (2014). Influence of Xuesaitong injection to forming of acute cerebral infarction. Chin. J. Exp. Trad. Med. Form., 196–200. 10.13422/j.cnki.syfjx.2014100196

[B6] Dai L. Zhang Y. Jiang Y. Chen K. (2022). Panax notoginseng preparation plus aspirin versus aspirin alone on platelet aggregation and coagulation in patients with coronary heart disease or ischemic stroke: a meta-analysis of randomized controlled trials. Front. Pharmacol. 13, 1015048. 10.3389/fphar.2022.1015048 36569332 PMC9768032

[B7] Dong X. Zheng L. Lu S. Yang Y. (2017). Neuroprotective effects of pretreatment of ginsenoside Rb1 on severe cerebral ischemia-induced injuries in aged mice: involvement of anti-oxidant signaling. Geriatr. Gerontol. Int. 17, 338–345. 10.1111/ggi.12699 26712031

[B8] Fisher M. Savitz S. I. (2022). Pharmacological brain cytoprotection in acute ischaemic stroke — renewed hope in the reperfusion era. Nat. Rev. Neurol. 18, 193–202. 10.1038/s41582-021-00605-6 35079135 PMC8788909

[B9] Goncalves A. Su E. J. Muthusamy A. Zeitelhofer M. Torrente D. Nilsson I. (2022). Thrombolytic tPA-induced hemorrhagic transformation of ischemic stroke is mediated by PKCβ phosphorylation of occludin. Blood 140, 388–400. 10.1182/blood.2021014958 35576527 PMC9335502

[B10] Guan Y. (2019). The effects of Xueshuantong injection combined with urokinase intravenous thrombolysis on neurological deficits and vWF, hs-CRP in patients with ischemic stroke. Chronic Pathol. J, 1012–1013+1016. 10.16440/j.cnki.1674-8166.2019.07.018

[B11] Guyatt G. H. Oxman A. D. Vist G. E. Kunz R. Falck-Ytter Y. Alonso-Coello P. (2008). GRADE: an emerging consensus on rating quality of evidence and strength of recommendations. BMJ 336, 924–926. 10.1136/bmj.39489.470347.AD 18436948 PMC2335261

[B12] Hu S. Wu Y. Zhao B. Hu H. Zhu B. Sun Z. (2018). Panax notoginseng saponins protect cerebral microvascular endothelial cells against oxygen-glucose deprivation/reperfusion-induced barrier dysfunction via activation of PI3K/Akt/Nrf2 antioxidant signaling pathway. Molecules 23, 2781. 10.3390/molecules23112781 30373188 PMC6278530

[B13] Huo J. Xie Z. (2018). Clinical study of Xueshuantong in combination with urokinase for the treatment of acute progressive ischemic stroke. Heilongjiang Med. J., 995–997. 10.14035/j.cnki.hljyy.2018.05.020

[B14] Li C. Xu T. Zhou P. Zhang J. Guan G. Zhang H. (2018). Post-marketing safety surveillance and re-evaluation of Xueshuantong injection. BMC Complement. Altern. Med. 18, 277. 10.1186/s12906-018-2329-z 30326892 PMC6192149

[B15] Li J. Wang H. (2024). Effect of Xueshuantong injection combined with alteplase thrombolytic therapy on ischemic stroke and its influences on cerebral vascular blood flow status, MCP-1 and VE-cadherin. Clin. Med. Res. Pract. 9, 72–76. 10.19347/j.cnki.2096-1413.202409018

[B16] Li Z. (2021). The impact of Xueshuantong injection in combination with alteplase on neurological function and vascular endothelial function in patients with acute ischemic stroke. Chin. Manip. Rehabil. Med., 42–44. 10.19787/j.issn.1008-1879.2021.16.017

[B17] Li Z. Fang R. (2023). The therapeutic effect of Xuesaitong injection combined with ateplase injection in the treatment of acute cerebral infarction and its impact on inflammatory factors. Clin. Ration. Drug Use 16, 48–51. 10.15887/j.cnki.13-1389/r.2023.30.013

[B18] Liu B. Li Y. Han Y. Wang S. Yang H. Zhao Y. (2021). Notoginsenoside R1 intervenes degradation and redistribution of tight junctions to ameliorate blood-brain barrier permeability by caveolin-1/mmp2/9 pathway after acute ischemic stroke. Phytomedicine 90, 153660. 10.1016/j.phymed.2021.153660 34344565

[B19] Liu J. (2015). Observing the efficacy of Xueshuantong injection in the treatment of stroke. Chin. J. Clin. Ration. Drug Use 67. 10.15887/j.cnki.13-1389/r.2015.08.043

[B20] Liu Q. Shi K. Wang Y. Shi F. (2023). Neurovascular inflammation and complications of thrombolysis therapy in stroke. Stroke 54, 2688–2697. 10.1161/STROKEAHA.123.044123 37675612

[B21] Liu S. Zou J. (2021). The effect of total saponins of Panax notoginseng on ischemia-reperfusion injury and hemorrhagic transformation in patients with acute ischemic stroke undergoing intravenous thrombolysis. Chin. J. Conval. Med. 30, 656–658. 10.13517/j.cnki.ccm.2021.06.033

[B22] Liu Z. Yue J. Li Y. (2022). Effect of xuesetong combined with alteplase on acute cerebral infarction and effect on serum levels of ET-1 and TXA2. Chin. Arch. Trad. Chin. Med., 213–216. 10.13193/j.issn.1673-7717.2022.08.051

[B23] Luo H. He M. Ni J. Liu J. Lan X. (2022). Clinical value and safety of thromboxane for injection plus intravenous thrombolysis with alteplase in the treatment of patients with acute cerebral infarction. Chin. Med Pharm 197–200. 10.3969/j.issn.2095-0616.2022.19.050

[B24] Ma Q. Li R. Wang L. Yin P. Wang Y. Yan C. (2021). Temporal trend and attributable risk factors of stroke burden in China, 1990–2019: an analysis for the global burden of disease study 2019. Lancet Public Health 6, e897–e906. 10.1016/S2468-2667(21)00228-0 34838196 PMC9047702

[B25] Mo X. (2019). Clinical observation of ateplase combined with Xueshuantong injection in the treatment of acute cerebral infarction. China Natur. 27, 62–63. 10.19621/j.cnki.11-3555/r.2019.1134

[B26] Moskowitz M. A. Lo E. H. Iadecola C. (2010). The science of stroke: mechanisms in search of treatments. Neuron 67, 181–198. 10.1016/j.neuron.2010.07.002 20670828 PMC2957363

[B27] National Institute of Neurological Disorders and Stroke rt-PA Stroke Study Group (1995). Tissue plasminogen activator for acute ischemic stroke. N. Engl. J. Med. 333, 1581–1587. 10.1056/NEJM199512143332401 7477192

[B28] Page M. J. McKenzie J. E. Bossuyt P. M. Boutron I. Hoffmann T. C. Mulrow C. D. (2021). The PRISMA 2020 statement: an updated guideline for reporting systematic reviews. BMJ 372, n71. 10.1136/bmj.n71 33782057 PMC8005924

[B29] Peng B. Liu M. Cui L. (2018). Chinese guidelines for diagnosis and treatment of acute ischemic stroke 2018. Chin. J. Neurol. 51, 666–682. 10.3760/cma.j.issn.1006-7876.2018.09.004

[B30] Phipps M. S. Cronin C. A. (2020). Management of acute ischemic stroke. BMJ 368, l6983. 10.1136/bmj.l6983 32054610

[B31] Powers W. J. Rabinstein A. A. Ackerson T. Adeoye O. M. Bambakidis N. C. Becker K. (2019). Guidelines for the early management of patients with acute ischemic stroke: 2019 update to the 2018 guidelines for the early management of acute ischemic stroke: a guideline for healthcare professionals from the American heart association/American stroke association. Stroke 50, e344–e418. 10.1161/STR.0000000000000211 31662037

[B32] Shi X. Feng L. Li Y. Qin M. Li T. Cheng Z. (2023). Efficacy and safety of Panax notoginseng saponins (Xuesaitong) for patients with acute ischemic stroke: a systematic review and meta-analysis of randomized controlled trials. Front. Pharmacol. 14, 1280559. 10.3389/fphar.2023.1280559 37908976 PMC10614024

[B33] Shi X. Yu W. Liu L. Liu W. Zhang X. Yang T. (2017). Panax notoginseng saponins administration modulates pro-/Anti-Inflammatory factor expression and improves neurologic outcome following permanent mcao in rats. Metab. Brain Dis. 32, 221–233. 10.1007/s11011-016-9901-3 27585466

[B34] Sterne J. A. C. Savović J. Page M. J. Elbers R. G. Blencowe N. S. Boutron I. (2019). RoB 2: a revised tool for assessing risk of bias in randomised trials. BMJ 366, l4898. 10.1136/bmj.l4898 31462531

[B35] Sun T. Wang P. Deng T. Tao X. Li B. Xu Y. (2020). Effect of Panax notoginseng saponins on focal cerebral ischemia-reperfusion in rat models: a meta-analysis. Front. Pharmacol. 11, 572304. 10.3389/fphar.2020.572304 33643030 PMC7908036

[B36] Tsao C. W. Aday A. W. Almarzooq Z. I. Anderson C. A. M. Arora P. Avery C. L. (2023). Heart disease and stroke statistics-2023 update: a report from the American heart association. Circulation 147, e93–e621. 10.1161/CIR.0000000000001123 36695182 PMC12135016

[B37] Tsivgoulis G. Katsanos A. H. Sandset E. C. Turc G. Nguyen T. N. Bivard A. (2023). Thrombolysis for acute ischaemic stroke: current status and future perspectives. Lancet Neurol. 22, 418–429. 10.1016/S1474-4422(22)00519-1 36907201

[B38] Tu W. Wang L. Special Writing Group of China Stroke Surveillance Report (2023). China stroke surveillance report 2021. Mil. Med. Res. 10, 33. 10.1186/s40779-023-00463-x 37468952 PMC10355019

[B39] Wang L. Zhao H. Zhai Z. Qu L. (2018). Protective effect and mechanism of ginsenoside Rg1 in cerebral ischaemia-reperfusion injury in mice. Biomed. Pharmacother. 99, 876–882. 10.1016/j.biopha.2018.01.136 29710487

[B40] Wang S. Li M. Guo Y. Li C. Wu L. Zhou X. (2017). Effects of Panax notoginseng ginsenoside Rb1 on abnormal hippocampal microenvironment in rats. J. Ethnopharmacol. 202, 138–146. 10.1016/j.jep.2017.01.005 28065779

[B41] Wu L. Song H. Zhang C. Wang A. Zhang B. Xiong C. (2023). Efficacy and safety of Panax notoginseng saponins in the treatment of adults with ischemic stroke in China: a randomized clinical trial. JAMA Netw. Open 6, e2317574. 10.1001/jamanetworkopen.2023.17574 37338907 PMC10282883

[B42] Xiao Q. Kang Z. Liu C. Tang B. (2022). Panax notoginseng saponins attenuate cerebral ischemia-reperfusion injury via mitophagy-induced inhibition of NLRP3 inflammasome in rats. Front. Biosci. Landmark Ed. 27, 300. 10.31083/j.fbl2711300 36472098

[B43] Xie H. (2013). Clinical effect and mechanism of Xuesaitong applied to cerebral infarction. Chin. Mod. Med. 61–62. 10.3969/j.issn.1674-4721.2013.15.029

[B44] XinYao J. KeYi W. JingBo Z. ChunXiang L. Hui W. Zhi L. (2020). Post-marketing intensive safety monitoring of Injection of Xuesaitong (lyophilized) in 30097 cases. Zhongguo Zhong Yao Za Zhi 45, 5029–5033. 10.19540/j.cnki.cjcmm.20200302.503 33350279

[B45] Xu Y. Tan H. Li S. Wang N. Feng Y. (2018). Panax notoginseng for inflammation-related chronic diseases: a review on the modulations of multiple pathways. Am. J. Chin. Med. 46, 971–996. 10.1142/S0192415X18500519 29976083

[B46] Yan D. Li Y. Zhang L. Zhu S. Duan J. (2020). The effects of xue-sai-tong injection combined with intravenous thrombolysis on hemodynamics, nerve function, serum hcy, nse and S-100β in patients with ischemic stroke. Pract. J. Clin. Med. 27–30. 10.3969/j.issn.1672-6170.2020.01.008

[B47] Zeng L. Yang S. Cui W. Ban Y. Gao C. Meng X. (2023). The therapeutic effect of Xueshuantong injection combined with ateplase in the treatment of acute ischemic cerebrovascular disease. Liaoning J. Tradit. Chin. Med., 1–9. 10.13192/j.issn.1000-1719.2024.04.022

[B48] Zhang G. Xia F. Zhang Y. Zhang X. Cao Y. Wang L. (2016). Ginsenoside Rd is efficacious against acute ischemic stroke by suppressing microglial proteasome-mediated inflammation. Mol. Neurobiol. 53, 2529–2540. 10.1007/s12035-015-9261-8 26081140

[B49] Zhang J. (2023). Effects of Xuesaitong for injection combined with alteplase intravenous thrombolysisand aspirin in treatment of patients with acute cerebral infarction. Med. J. Chin. People Health, 79–82. 10.3969/j.issn.1672-0369.2023.14.024

[B50] Zhang J. Guo F. Zhou R. Xiang C. Zhang Y. Gao J. (2021). Proteomics and transcriptome reveal the key transcription factors mediating the protection of Panax notoginseng saponins (PNS) against cerebral ischemia/reperfusion injury. Phytomedicine 92, 153613. 10.1016/j.phymed.2021.153613 34500302

[B51] Zhang Y. Wang Y. Song Z. Li R. Wang Y. (2022). The effect of butylphthalide combined with Xuesaitong injection on oxidative stress and cerebral hemodynamics in early acute cerebral infarction patients. Chin. J. Difficult Diffic. Cases 21, 247–251. 10.3969/j.issn.1671-6450.2022.03.006

[B52] Zhao F. Ma Y. Wang W. (2024). Effect of Xueshuantong combined with alteplase in the treatment of acute cerebral infarction and its influence on cerebral hemodynamics. Clin. Med. Res. Pract. 9, 66–69+82. 10.19347/j.cnki.2096-1413.202403017

