# Supplementary material for: The use of Panax notoginseng saponins injections after intravenous thrombolysis in acute ischemic stroke: a systematic review and meta-analysis
Source: Front Pharmacol. 2024 Jun 5;15:1376025. doi: 10.3389/fphar.2024.1376025 (PMC11185952; doi:10.3389/fphar.2024.1376025)
Supplement: Supplementary file 1 [file DataSheet1.docx]

Supplementary Material

# Diagnostic criteria

The diagnostic criteria for acute ischemic stroke were formulated based on the *Chinese Guidelines for Diagnosis and Treatment of Acute Ischemic Stroke in 2018* and the *2018 Guidelines for the Early Management of Acute Ischemic Stroke from the American Heart Association/American Stroke Association*.

Diagnostic criteria:

1) Acute onset of symptoms. 2) Focal neurological deficits, such as unilateral facial or limb weakness or numbness, language impairment, with a few presenting comprehensive neurological deficits. 3) Presence of responsible lesions on imaging or symptoms/signs persisting for more than 24 hours. 4) Exclusion of non-vascular causes. 5) Brain CT/MRI ruling out cerebral hemorrhage.

# Detailed information for *Panax notoginseng* saponins injections

*Panax notoginseng* saponins injections mainly consists of two types, Xuesaitong injection and Xueshuantong injection, as presented in Table 1.

**Table 1** Detailed information for *Panax notoginseng* saponins injections

| Type | Ingredients | Specifications | Usage and dosage | Indication |
| --- | --- | --- | --- | --- |
| Xuesaitong injection | *Panax notoginseng* saponins. The main components are ginsenoside Rb1, ginsenoside Rg1, notoginsenoside R1, ginsenoside Rd, and ginsenoside Re | 2ml: 100mg;  2ml: 200mg;  5ml: 250mg;  10ml: 250mg;  10ml: 400mg | Intravenous drip, 200~400mg at a time, diluted in 250~500ml of 5~10% glucose injection and administered slowly. Once a day | Used for hemiplegia after a stroke, atherosclerotic thrombotic cerebral infarction, cerebral embolism, central retinal vein occlusion |
| Xueshuantong injection |  | 2ml: 70mg;  5ml: 175mg | Intravenous drip, 2~5ml at a time, diluted in 250~500ml of 10% glucose injection. Once or twice a day | Dilates blood vessels, improves blood circulation. Used for central retinal vein occlusion, sequelae of cerebrovascular diseases, intraocular diseases, and anterior chamber bleeding in the eye |

# Search Queries

The search queries for China National Knowledge Infrastructure Database (CNKI), VIP Database for Chinese Technical Periodicals (VIP), Wanfang Database (Wanfang), Chinese Biomedical Literature Database (SinoMed), Web of Science, MEDLINE, Embase, and Cochrane Library are shown in Table 2.

**Table 2** Search queries for each database

| Database | Search query |
| --- | --- |
| CNKI | SU=("血塞通" + "血塞通注射液" + "血栓通" + "血栓通注射液") AND SU=("脑卒中" + "卒中" + "脑梗死" + "脑梗" + "中风" + "急性脑血管病") AND SU=("临床"+"疗效"+"随机"+"观察"+"评价"+"安全性"+"有效性") |
| VIP | M=("血塞通"+"血塞通注射液"+"血栓通"+"血栓通注射液") AND M=("脑卒中"+"卒中"+"脑梗死"+"脑梗"+"中风"+"急性脑血管病") AND M=("临床"+"疗效"+"随机"+"观察"+"评价"+"安全性"+"有效性") |
| Wanfang | 主题:("血塞通" OR "血塞通注射液" OR "血栓通" OR "血栓通注射液") AND 主题:("脑卒中" OR "卒中" OR "脑梗死" OR "脑梗" OR "中风" OR "急性脑血管病") AND 主题:("临床" OR "疗效" OR "随机" OR "观察" OR "评价" OR "安全性" OR "有效性") |
| SinoMed | ("血塞通"[标题:智能] OR "血塞通注射液"[标题:智能] OR "血栓通"[标题:智能] OR "血栓通注射液"[标题:智能]) AND ("卒中"[标题:智能] OR "脑梗"[标题:智能] OR "中风"[标题:智能] OR "急性脑血管病"[标题:智能]) AND ("临床"[标题:智能] OR "疗效"[标题:智能] OR "随机"[标题:智能] OR "观察"[标题:智能] OR "评价"[常用字段:智能] OR "安全性"[常用字段:智能] OR "有效性"[常用字段:智能]) |
| Web of Science | TS=(Cerebral Infarction OR Infarction, Cerebral OR Cerebral Infarct OR Infarct, Cerebral OR Stroke OR Cerebrovascular Accident OR Apoplexy OR Cerebrovascular Accident, Acute OR Acute Cerebrovascular Accident) AND TS=(*Panax notoginseng* OR Xuesetong OR Xuesaitong OR Xue sai tong OR Xueshuantong OR Xue shuan tong OR Xuesaitong Injection OR Xueshuantong Injection) AND TS=(randomized controlled trial OR randomized clinical trial OR randomized trial OR clinical trial OR randomized controlled trial OR randomized clinical trial OR randomized trial OR clinical trial) |
| MEDLINE | #1 "Cerebral Infarction"[Mesh Terms] OR "Infarction, Cerebral"[Title/Abstract] OR "Cerebral Infarct"[Title/Abstract] OR "Infarct, Cerebral"[Title/Abstract] OR "Stroke"[Mesh Terms] OR "Cerebrovascular Accident"[Title/Abstract] OR "Apoplexy"[Title/Abstract] OR "Cerebrovascular Accident, Acute"[Title/Abstract] OR "Acute Cerebrovascular Accident"[Title/Abstract]  #2 "*Panax notoginseng*"[Mesh Terms] OR "Xuesetong"[Title/Abstract] OR "Xuesaitong"[Title/Abstract] OR "Xue sai tong"[Title/Abstract] OR "Xueshuantong"[Title/Abstract] OR "Xue shuan tong"[Title/Abstract] OR "Xuesaitong Injection"[Title/Abstract] OR "Xueshuantong Injection"[Title/Abstract]  #3 "randomized controlled trial"[Publication type] OR randomized clinical trial[Publication type] OR randomized trial[Publication type] OR clinical trial[Publication type] OR "randomized controlled trial"[Title/Abstract] OR randomized clinical trial[Title/Abstract] OR randomized trial[Title/Abstract] OR clinical trial[Title/Abstract]  #4 #1 AND #2 AND #3 |
| Embase | ('brain infarction'/exp OR 'cerebrovascular accident'/exp OR 'Cerebral Infarction':ti,ab,kw OR 'Cerebral Infarct':ti,ab,kw OR 'Infarct, Cerebral':ti,ab,kw OR 'Stroke':ti,ab,kw OR 'Apoplexy':ti,ab,kw) AND ('*Panax notoginseng* extract'/exp OR 'Xuesetong':ti,ab,kw OR 'Xuesaitong':ti,ab,kw OR 'Xue sai tong':ti,ab,kw OR 'Xueshuantong':ti,ab,kw OR 'Xue shuan tong':ti,ab,kw OR 'Xuesaitong Injection':ti,ab,kw OR 'Xueshuantong Injection':ti,ab,kw) AND ('randomized controlled trial':ti,ab,kw OR 'randomized clinical trial':ti,ab,kw OR 'randomized trial':ti,ab,kw) |
| Cochrane Library | "Cerebral Infarction" OR "Infarction, Cerebral" OR "Cerebral Infarct" OR "Infarct, Cerebral" OR "Stroke" OR "Cerebrovascular Accident" OR "Apoplexy" OR "Cerebrovascular Accident, Acute" OR "Acute Cerebrovascular Accident" in Title Abstract Keyword AND "*Panax notoginseng*" OR "Xuesetong" OR "Xuesaitong" OR "Xue sai tong" OR "Xueshuantong" OR "Xue shuan tong" OR "Xuesaitong Injection" OR "Xueshuantong Injection" in Title Abstract Keyword - in Trials (Word variations have been searched) |

Abbreviation: CNKI, China National Knowledge Infrastructure Database, VIP, VIP Database for Chinese Technical Periodicals, Wanfang, Wanfang Database, SinoMed, Chinese Biomedical Literature Database

# Rules for Assessment of the Certainty of Evidence

The assessment of certainty of evidence was divided into five elements: study design, inconsistency, indirectness, imprecision, and publication bias, each with their respective criteria for downgrade as shown in Table 3.

**Table 3** Rules for assessment of the certainty of evidence

| Element | Criteria for downgrade |
| --- | --- |
| Study design | If the majority of the information based on the revised Cochrane Risk of Bias tool assessment was rated as moderate, the evidence was downgraded by one level. If the majority was rated as high, the evidence was downgraded by two levels |
| Inconsistency | If heterogeneity tests showed I^2^ exceeding 75%, the evidence was downgraded by two levels. If I^2^ was exceeding 50% and less than 75%, the evidence was downgraded by one level |
| Indirectness | Assessment included several components: Population differences, Intervention differences, Outcome measurement differences, and Indirect comparisons. If there was a serious suspicion regarding the directness of the evidence, the evidence was downgraded by one level. If there was a very serious suspicion regarding the directness of the evidence, the evidence was downgraded by two levels |
| Imprecision | If the 95% confidence interval crossed the null line, the evidence was downgraded by one level. If the total sample size of included studies was less than 400, the evidence was downgraded by one level |
| Publication bias | If there was publication bias, the evidence was downgraded by one level |

# The basic information and access methods of the original literature

Here are the basic information, DOIs, and English abstracts of the literature included in the study. If the literature does not have a DOI, its URLs will be provided to facilitate readers' online access.

**1. Bian, H. (2019). The Impact and Mechanism Study of Xueshuantong Combined with Alteplase Treatment on the Neurological Function of Patients with Acute Ischemic Stroke. J Health must-Read, 221.**

**URL:** <https://d.wanfangdata.com.cn/periodical/ChlQZXJpb2RpY2FsQ0hJTmV3UzIwMjMxMjI2EhBqa2JkMDAxMjAxOTIwMjU3Gghld3NzZnFhaQ%3D%3D>

**Abstract:**

Objective: To investigate the effects of Xueshuantong combined with ateplase therapy on neurological function in patients with acute cerebral infarction and explore the underlying mechanisms. Method: 70 patients with acute cerebral infarction admitted to our hospital from April 2017 to February 2019 were randomly divided into a study group and a control group using statistical methods. The study group treated patients with Xueshuantong combined with ateplase, while the control group treated with ateplase. The corresponding changes in indicators were compared between the two groups. Result: Before treatment, the corresponding indicators of both groups of patients did not reach a significant level (P>0.05). After treatment, the S-100B, NSE, NIHSS score, Hcy, and sICAM-1 in the study group were significantly reduced compared to the control group, while CGRP, NGF, MVP, PLT, and PDW were significantly increased compared to the control group (P<0.05). Conclusion: The use of Xueshuantong combined with ateplase in the treatment of acute cerebral infarction patients can significantly improve their neurological function. The possible reason for this may be the impact on platelet function and the improvement of cytokine secretion.

**2. Chen, Y. (2013). Xueshuantong Clinical Observation on the Treatment of the Acute Phase of Ischemic Stroke. Asian Pac Trad Med, 157–158.**

**URL:** <https://kns.cnki.net/kcms2/article/abstract?v=gPw7xOyBVLF0f5YIuggkQYGIENh7YezB-qHjFKD9XlVotkHY2Es-llfGtgz22spmo0nHXcq0OFt0GrNIHu0hsoTrzubFzhtEdHTHuwEI3XSVYRrXcEt1NMCrha71sKcpbPEPsvLEPAk=&uniplatform=NZKPT&language=CHS>

**Abstract:**

Objective: To observe the clinical efficacy of Xueshuantong Injection in the treatment of acute ischemic stroke. Method: 150 patients with acute ischemic stroke were randomly divided into an observation group of 80 cases and a control group of 70 cases. Both groups were treated with conventional Western medicine thrombolysis. On this basis, the observation group received intravenous infusion of Xueshuantong injection for 3 consecutive days as a course of treatment, and their clinical efficacy was compared. Result: After 72 hours of treatment, the total effective rate of the observation group was significantly higher than that of the control group (P<0.01), with statistical significance. After treatment, there were significant differences in whole blood viscosity, erythrocyte sedimentation rate, and whole blood reduction viscosity compared to before treatment (P<0.05), but there was no significant difference between groups before and after treatment (P>0.05). There was no significant difference in NIHSS scores between the two groups of patients before treatment (P>0.05). After treatment, NIHSS scores improved significantly in both groups, but the observation group showed significant improvement compared to the control group (P<0.01), with statistical significance. Conclusion: The application of Xueshuantong Injection in the acute phase of ischemic stroke can significantly improve neurological function, promote the disappearance of clinical symptoms and signs, and is worthy of clinical promotion and application.

**3. Chen, Z., and Chen, H. (2014). Analysis of Influential Factors on the Efficacy of Xuesaitong Injection in Treating Patients with Ischemic Stroke. J Hainan Med, 2575–2577. doi: 10.3969/j.issn.1003-6350.2014.17.1005**

**Abstract:**

Objective: To explore the influencing factors of the clinical efficacy of Xuesaitong injection in the treatment of cerebral infarction. Method: 160 patients with cerebral infarction admitted to the Department of Neurology of our hospital from January 2012 to January 2013 were randomly divided into a treatment group and a control group, with 80 cases in each group. Both groups of patients received the same basic comprehensive treatment, and the treatment group received traditional Chinese medicine Xuesaitong injection on the basis of comprehensive treatment. The National Institutes of Health Stroke Scale (NIHSS) was used to evaluate the degree of neurological deficits before and after treatment in two groups of patients. Collect relevant indicators that may affect the efficacy of Xuesaitong in treating cerebral infarction, and use multivariate logistic regression analysis to identify the risk factors that affect the efficacy. Result: There was no statistically significant difference in NIHSS scores between the two groups of patients before treatment (P>0.05). Both groups showed improvement in NIHSS scores after treatment (P<0.05), and the treatment group had better NIHSS scores than the control group after treatment (P<0.05). The total effective rate of the treatment group (82.5%) was also higher than that of the control group (67.5%) (P<0.05). Multivariate logistic regression analysis showed that diabetes, onset to treatment time>4h were independent risk factors affecting the efficacy of Xuesaitong in the treatment of cerebral infarction. Conclusion: Xuesaitong Injection is an effective drug for treating cerebral infarction. Diabetes and the time from onset to treatment>4h were independent risk factors affecting the efficacy of xuesaitong in the treatment of cerebral infarction.

**4. Cheng, M., Li, H., and Qi, S. (2014). Influence of Xuesaitong Injection to Forming of Acute Cerebral Infarction. Chinese J Exp Trad Med Form, 196–200. doi: 10.13422/j.cnki.syfjx.2014100196**

**Abstract:**

Objective: To explore the therapeutic effect of Xuesaitong injection on acute cerebral infarction and its impact on thrombosis formation, as well as its mechanism of action. Method: 63 patients with acute cerebral infarction were randomly divided into a control group of 30 cases and an observation group of 33 cases. Both groups were treated with corresponding internal medicine according to the Chinese Guidelines for the Diagnosis and Treatment of Acute Ischemic Stroke 2010. Anticoagulant use is aspirin enteric coated tablets, 100 mg/time, once a day, taken after dinner. The observation group received injection of Xuesaitong in addition to the control group's treatment. 0.4 g/time, intravenous infusion, once a day. Both treatment groups have a duration of 14 days. The severity of neurological deficits was evaluated using the National Institutes of Health Stroke Scale (NIHSS) on day 7 and 14, respectively. Detect changes in anticoagulant enzyme III (AT III), fibrinogen (FIB), D-dimer (D-D), platelet count, platelet volume distribution width (PDW), mean platelet volume (MPV), large platelet ratio (PLCR), platelet aggregation rate (MPAR), thromboxane B (TXB), and 6-keto prostaglandin Fla (6-Keto PGF-la) before and after treatment. Result: According to analysis, the clinical efficacy of the observation group was better than that of the control group (P<0.05). After 7 days of treatment, the NIHSS scores of both groups decreased compared to before treatment (P<0.01). 14 days after treatment, the NIHSS scores of both groups continued to decrease (P<0.01), and the NIHSS scores of the observation group were lower than those of the control group (P<0.01). After treatment, FIB, D-D, and MPAR in the observation group were lower than those in the control group (P<0.01), while AT III was higher than that in the control group (P<0.01). After treatment, the PDW and MPV of the observation group decreased compared to before treatment, and the P-LCR decreased compared to before treatment. Compared with the control group after treatment, the difference was also statistically significant (P<0.05 or P<0.01). After treatment, the level of TXB in the observation group was lower than that in the control group, and the level of 6-Keto PGF-la was higher than that in the control group (P<0.01). Conclusion: Injection of Xuesaitong can improve blood supply in the infarcted area and surrounding penumbra of acute cerebral infarction patients through multiple pathways of antiplatelet aggregation and thrombus inhibition, promoting the recovery of neurological function.

**5. Guan, Y. (2019). The Effects of Xueshuantong Injection Combined with Urokinase Intravenous Thrombolysis on Neurological Deficits and vWF, hs-CRP in Patients with Ischemic Stroke. Chronic Pathol J, 1012-1013+1016. doi: 10.16440/j.cnki.1674-8166.2019.07.018**

**Abstract:**

Objective: To explore the effect of Xueshuantong injection combined with intravenous thrombolysis with urokinase on neurological deficits in patients with cerebral infarction. Method: 78 patients with cerebral infarction admitted to Yanhu District People's Hospital in Yuncheng City from July 2016 to October 2018 were selected and randomly divided into a control group and an observation group using a simple randomization method, with 39 cases in each group. Among them, the control group received basic treatment and intravenous thrombolysis with urokinase, while the observation group received treatment with Xueshuantong injection on the basis of the control group's treatment. The efficacy observation period of both groups was 15 days. The total effective rate, serum levels of von Willebrand factor (vWF) and high-sensitivity C-reactive protein (hs CRP) were compared between the two groups after treatment, as well as the National Institutes of Health Stroke Scale (NIHSS) score. Result: After treatment, the total effective rate of the observation group was 89.74% higher than that of the control group, which was 69.23% (P<0.05). The NIHSS score, hs CRP, and vWF levels were lower than before treatment, and all were lower than those of the control group (P<0.05). During the treatment period, neither group experienced any adverse reactions such as bleeding. Conclusion: The combination of Xueshuantong injection and intravenous thrombolysis with urokinase can inhibit platelet activation and aggregation, alleviate inflammatory reactions, improve neurological deficits, and is safe and reliable in the treatment of cerebral infarction.

**6. Huo, J., and Xie, Z. (2018). Clinical Study of Xueshuantong in Combination with Urokinase for the Treatment of Acute Progressive Ischemic Stroke. Heilongjiang Med J, 995–997. doi: 10.14035/j.cnki.hljyy.2018.05.020**

**Abstract:**

Objective: To analyze the clinical advantages of the combination of Xueshuantong and Urokinase therapy in the treatment of acute progressive cerebral infarction. Method: 120 patients with acute progressive cerebral infarction were selected in this article, all located in the undergraduate department of our hospital. The method was statistical randomization, and the grouping was done by drawing lots to ensure randomness. The grouping results were the study group and the control groups A and B groups. The control groups A and B were treated separately with drugs, including urokinase and thromboxane. The study group was treated with a combination of xueshuantong and urokinase, and the improvement of symptoms and signs was compared. Result: In terms of treatment effectiveness, cure rate, and avoidance of adverse reactions, the research group has significant advantages, with significant differences compared to control groups A and B, P<0.05. Moreover, imaging and hemodynamic tests are indispensable in the analysis of clinical efficacy, and there is a significant difference between the two groups, with P<0.05. Conclusion: For patients with cerebral infarction, the use of Xueshuantong combined with urokinase therapy can effectively improve symptoms and signs, better protect nervous system function, have mild side effects, and have a good prognosis. It is recommended to promote clinical application.

**7. Li J., and Wang H. (2024). Effect of Xueshuantong injection combined with alteplase thrombolytic therapy on ischemic stroke and its influences on cerebral vascular blood flow status, MCP-1 and VE-cadherin. Clin Med Res Pract 9, 72–76. doi: 10.19347/j.cnki.2096-1413.202409018**

**Abstract:**

Objective: To explore the effect of Xueshuantong injection combined with ateplase thrombolysis in the treatment of ischemic stroke, and its influence on cerebral vascular blood flow status, monocyte chemotactic protein-1 (MCP-1), and vascular endothelial cell cadherin (VE cadherin). Method: 100 patients with ischemic stroke admitted to our hospital from March 2020 to March 2022 were selected as the study subjects. They were randomly divided into a control group and an observation group using a random number table method, with 50 cases in each group. After admission, both groups of patients received routine treatment, while the control group received thrombolytic therapy with injection of ateplase. The observation group received treatment with thrombus injection in addition to the control group. Compare the therapeutic effects of two groups. Result: The total effective rate of the observation group was 94.00%, which was higher than the control group's 78.00% (P<0.05). After treatment, the blood flow velocities of the bilateral middle cerebral artery (MCA), anterior cerebral artery (ACA), posterior cerebral artery (PCA), and left and right vertebral artery (VA) in the observation group were all higher than those in the control group (P<0.05). After treatment, the levels of neuron specific enolase (NSE) in the observation group were lower than those in the control group, while the levels of nerve growth factor (NGF) and brain-derived neurotrophic factor (BDNF) were higher than those in the control group (P<0.05). After treatment, the levels of MCP-1 and VE cadherin in the observation group were lower than those in the control group (P<0.05). Conclusion: The combination of Xueshuantong Injection and Ateplase thrombolysis in the treatment of ischemic stroke can not only improve clinical efficacy and cerebral vascular blood flow status, but also regulate MCP-1 and VE cadherin levels, promote neurological function recovery, and is worth promoting.

**8. Li, Z. (2021). The Impact of Xueshuantong Injection in Combination with Alteplase on Neurological Function and Vascular Endothelial Function in Patients with Acute Ischemic Stroke. Chinese Manip Rehabil Med, 42–44. doi: 10.19787/j.issn.1008-1879.2021.16.017**

**Abstract:**

Objective: To explore the effects of Xueshuantong Injection combined with ateplase on neurological and endothelial function in patients with acute cerebral infarction. Method: Ninety patients with acute cerebral infarction who were hospitalized in the Neurology Department of Jianyang People's Hospital from December 2017 to December 2019 were selected and randomly divided into an observation group and a control group, with 45 cases in each group, using a random number table method. Both groups received comprehensive treatment after admission. The control group was injected with ateplase, while the observation group was given Xueshuantong injection in addition to the control group. Compare the NIHSS and BI scores of two groups of patients before and after treatment; Neuroinjury related factors: NSE, S100B protein, and BDNF; Factors related to vascular endothelial function: ET-1, PAO, VEGF. Result: After treatment, the NIHSS score of the observation group was lower than that of the control group (P<0.05), and the BI score was higher than that of the control group (P<0.05). After treatment, the NSE and S100B of the observation group were lower than those of the control group, and the BDNF was higher than that of the control group (P<0.05). After treatment, the levels of ET-1 and PAO in the observation group were lower than those in the control group, while the levels of VEGF were higher than those in the control group (P<0.05). Conclusion: The combination of Xueshuantong Injection and Ateplase can significantly improve the neurological and endothelial function of patients with acute cerebral infarction.

**9. Li, Z., and Fang, R. (2023). The therapeutic effect of Xuesaitong injection combined with ateplase injection in the treatment of acute cerebral infarction and its impact on inflammatory factors. Clin Ration Drug Use 16, 48–51. doi: 10.15887/j.cnki.13-1389/r.2023.30.013**

**Abstract:**

Objective: To observe the therapeutic effect of Xuesaitong Injection combined with Ateplase Injection in the treatment of acute cerebral infarction (ACI) and its impact on inflammatory factors. Method: 86 ACI patients admitted to Guixi People's Hospital from February 2022 to April 2023 were divided into a control group and an experimental group, with 43 patients in each group, using a random number table method. The control group was given ateplase injection, while the experimental group was given Xuesaitong injection on the basis of the control group for 10 days of treatment. Compare the clinical efficacy of two groups, serum inflammatory factors [interleukin-6 (IL-6), C-reactive protein (CRP), tumor necrosis factor- α (TNF)- α)] before and after 10 days of treatment, Cerebral hemodynamic indicators [middle cerebral artery pulsatility index (PI), resistance index (RI), mean blood flow velocity (Vm)], Barthel index evaluation scale (BI), stroke specific quality of life scale (SS-00L) score, adverse reactions. Results: The total effective rate of treatment in the experimental group was higher than that in the control group. After 10 days of treatment, serum IL-6, CRP, TNF-α in both groups were measured The level decreased compared to before medication, and the experimental group was lower than the control group (P<0.01). The PI and RI of the middle cerebral artery in two groups decreased compared to before medication, while the Vm of the middle cerebral artery increased compared to before medication, and the decrease/increase amplitude in the experimental group was greater than that in the control group (P<0.05 or P<0.01). Two groups of BI and SS-00L showed an increase in scores compared to before medication, and the experimental group was higher than the control group (P<0.01). There was no statistically significant difference in the total incidence of adverse reactions between the experimental group and the control group during treatment (6.98% vs. 4.65%, P=1.00). Conclusion: The combination of Xuesaitong Injection and Ateplase Injection has a better therapeutic effect on ACI, which can reduce serum inflammatory factor levels, alleviate inflammatory reactions, improve cerebral hemodynamics, promote neurological function recovery, improve patients' daily living ability and quality of life, and is relatively safe.

**10. Liu, J. (2015). Observing the Efficacy of Xueshuantong Injection in the Treatment of Stroke. Chinese J Clin Ration Drug Use, 67. doi: 10.15887/j.cnki.13-1389/r.2015.08.043**

**Abstract:**

Objective: To observe the clinical efficacy of Xueshuantong injection in the treatment of stroke. Method: 80 middle-aged and elderly stroke patients admitted to the hospital were randomly divided into an observation group and a control group, with 40 cases in each group. The observation group received injection of Xueshuantong freeze-dried powder in addition to conventional treatment, while the control group received conventional treatment. Result: The total effective rate of the observation group was 90.0%, which was significantly higher than the control group's 72.5%, and the difference was statistically significant (P<0.05). Conclusion: This study demonstrates that the injection of Xueshuantong has high clinical application value for stroke in middle-aged and elderly patients, providing a basis for better prevention and treatment of cerebral infarction in clinical practice, and is worthy of promotion and popularization.

**11. Liu S., and Zou J. (2021). The effect of total saponins of Panax notoginseng on ischemia-reperfusion injury and hemorrhagic transformation in patients with acute ischemic stroke undergoing intravenous thrombolysis. Chin J Convalescent Med 30, 656–658. doi: 10.13517/j.cnki.ccm.2021.06.033**

**Abstract:**

Objective: To explore the effect of total saponins of Panax notoginseng on ischemia-reperfusion injury and hemorrhagic transformation (HT) in patients with acute ischemic stroke undergoing intravenous thrombolysis. Method: 90 patients with acute ischemic stroke admitted to a certain hospital from January 2018 to December 2019 were selected and randomly divided into two groups, each with 45 cases, according to a random number table. The control group received intravenous thrombolysis with rt PA, and on this basis, the treatment group was treated with a combination of total saponins of Panax notoginseng. Compare the degree of ischemia-reperfusion injury, neurological deficits, incidence of HT, and incidence of adverse reactions between two groups. Result: After treatment, the superoxide dismutase (SOD) level in the treatment group was higher than that in the control group, while the levels of malondialdehyde (MDA), matrix metalloproteinase-9 (MMP-9), fibronectin (FN), National Institutes of Health Stroke Scale (NIHSS) score, and incidence of HT were lower than those in the control group, with statistical significance (P<0.05). There was no statistically significant difference in the incidence of adverse reactions between the two groups (P>0.05). Conclusion: The application of total saponins of Panax notoginseng in patients with acute ischemic stroke undergoing intravenous thrombolysis can alleviate ischemia-reperfusion injury, alleviate neurological deficits, reduce the incidence of HT, and has high safety.

**12. Liu, Z., Yue, J., and Li, Y. (2022). Effect of Xuesetong Combined with Alteplase on Acute Cerebral Infarction and Effect on Serum Levels of ET-1 and TXA2. Chinese Arch Trad Chinese Med, 213–216. doi: 10.13193/j.issn.1673-7717.2022.08.051**

**Abstract:**

Objective: To investigate the efficacy of Xuesaitong combined with alteplase in the treatment of acute cerebral infarction and its effect on serum levels of ET-1 and TXA2. Methods: Eighty-four cases of patients with acute cerebral infarction admitted to the hospital were selected and randomly divided into control group (44 cases) and observation group (40 cases). The two groups were given routine treatment such as anti-platelet aggregation, stable atherosclerotic plaque and brain protection. The control group was given intravenous injection of alteplase. According to the condition and the weight of patients, the dosage was 0.6 mg/kg or 0.9 mg/kg, which was no more than 90 mg. First, 10% of the total amount was injected by intravenous injection, and the remaining was injected by 1 h intravenous pump. The observation group was given xuesaitong for injection on the basis of the control group. The recommended dose was 200 mg/time, dissolved in 0.996 normal saline 250 ml intravenously once a day. Both groups were treated for 10 days. The clinical efficacy, Barthel index (BI index) score, Modified Rankin Scale (MRS) score, National Institute of Health stroke scale (NlHSS) score, TCM syndrome score, serum levels of C-reactive protein (CRP), interleukin 6 (IL-6), tumor necrosis factor-α (TNF-α), endothelin-1 (ET-1), thromboxane A2 (TXA2) and hemodynamic indexes of the two groups were compared. Results: After treatment, the total effective rate in the observation group was higher (P < 0.05). Before treatment there were no significant differences in Bl index score, MRS score or NlHSS score between the two groups (P > 0.05). After treatment, Bl index scores in two groups were significantly increased, while MRS and NlHSS scores were significantly decreased (P < 0.05). The improvement of the observation group was significant (P < 0.05). After treatment, the TCM syndrome score and serum inflammatory factor levels were significantly decreased in both groups (P < 0.05). And those in the observation group were significantly decreased (P < 0.05). There were no significant differences in serum levels of ET-1 or TXA2 between the two groups before treatment (P > 0.05). After treatment, the serum levels of ET-1 and TXA2 in the two groups were significantly decreased (P < 0.05). And the levels of the observation group were more significant (P < 0.05). There were no significant differences in serum levels of hematocrit (HCT), whole blood viscosity (WBV), fibrinogen (FlB) or plasma viscosity (PV) between the two groups before treatment (P > 0.05). After treatment, the serum levels of HCT, WBV, FlB and PV in two groups were significantly decreased (P < 0.05). And those of the observation group were decreased significantly (P < 0.05). Conclusion: Xuesaitong combined with alteplase in the treatment of acute cerebral infarction has a good clinical effect, and can reduce serum levels of ET-1 and TXA2.

**13. Luo, H., He, M., Ni, J., Liu, J., and Lan, X. (2022). Clinical Value and Safety of Thromboxane for Injection Plus Intravenous Thrombolysis with Alteplase in the Treatment of patients with Acute Cerebral Infarction. Chinese Med and Pharm, 197–200.**

**URL:** <https://kns.cnki.net/kcms2/article/abstract?v=gPw7xOyBVLHQhgMFwA_UZnKs1fXV99Bf3P_nYh0liQ6jik45cpOLB4Af0iRixcoi82wG8-X5AvOxaJFAihJNPgEl8BoJB4vI_3EWAwz9BrDozf0AF-17zXg7itaYjzCFvnWl2G3e0vYVC6taRmvgmQ==&uniplatform=NZKPT&language=CHS>

**Abstract:**

Objective: To investigate the efficacy and safety of Xueshuantong injection combined with ateplase intravenous thrombolysis in patients with acute cerebral infarction. Method: Sixty patients with acute cerebral infarction who underwent treatment in the emergency department of Foshan Traditional Chinese Medicine Hospital from January to December 2021 were randomly divided into a control group (ateplase) and an experimental group (Xueshuantong Injection+ateplase) using a random number table method, with 30 cases in each group. Compare the thrombolytic effects, intracranial hemodynamic and biochemical indicators, improvement in neurological deficits, and drug-related adverse reactions between two groups. Result: The effective rate of thrombolysis treatment in the experimental group was higher than that in the control group, and the difference was statistically significant (P<0.05). Before thrombolysis treatment, there was no statistically significant difference in the peak systolic flow velocity (VP), end diastolic flow velocity (Vd), and mean flow velocity (Vm) between the two groups of intracranial hemodynamic indicators (P>0.05). After thrombolysis treatment, VP, Vd, and Vm increased in both groups, and the experimental group was higher than the control group, with statistical significance (P<0.05). Before thrombolytic therapy, there was no statistically significant difference in the blood biochemical indicators of neuron specific enolase (NSE), homocysteine (Hcy), and central nervous system specific protein (S-100B) between the two groups (P>0.05). After thrombolysis treatment, NSE, Hey, and S-100B in both groups decreased, and the experimental group was lower than the control group, with statistical significance (P<0.05). Before thrombolytic therapy, there was no statistically significant difference in NIHSS scores between the two groups for neurological deficits (P>0.05). After thrombolysis treatment, the NIHSS scores of both groups decreased, and the experimental group had fewer scores than the control group, with a statistically significant difference (P<0.05). The total incidence of drug-related adverse reactions in the experimental group was higher than that in the control group, but the difference was not statistically significant (P>0.05). Conclusion: The combination of Xueshuantong Injection and Ateplase intravenous thrombolysis has good clinical efficacy and high safety in the treatment of acute cerebral infarction.

**14. Mo, X. (2019). Clinical observation of ateplase combined with Xueshuantong injection in the treatment of acute cerebral infarction. China Natur 27, 62–63. doi: 10.19621/j.cnki.11-3555/r.2019.1134**

**Abstract:**

Objective: To observe the clinical effect of ateplase combined with Xueshuantong injection in the treatment of patients with acute cerebral infarction. Method: 70 patients with acute cerebral infarction were randomly divided into a control group and an observation group, with 35 cases in each group. The control group received routine treatment, while the observation group received combined treatment with Xueshuantong Injection on the basis of routine treatment. Compare the National Institutes of Health Stroke Scale (NIHSS) scores, serum levels of superoxide dismutase (SOD), malondialdehyde (MDA), catalase (CAT), and clinical efficacy between two groups before and after treatment. Result: Before treatment, there was no statistically significant difference in NIHSS score, SOD, MDA, and CAT between the two groups (P>0.05). After treatment, the NIHSS score and MDA in the observation group were significantly lower than those in the control group, while SOD and CAT were significantly higher than those in the control group, with statistical significance (P<0.05). The total effective rate of the observation group was 94.29%, significantly higher than the control group's 71.43%, and the difference was statistically significant (P<0.05). Conclusion: The use of ateplase and Xueshuantong injection in the treatment of acute cerebral infarction patients can significantly regulate various physiological indicators, improve neurological function scores, and have good clinical effects.

**15. Xie, H. (2013). Clinical Effect and Mechanism of Xuesaitong Applied to Cerebral Infarction. Chinese Mod Med, 61–62.**

**URL:** <https://kns.cnki.net/kcms2/article/abstract?v=gPw7xOyBVLEp6xnhnkyD1my4Kcl7yNIDe7saj9iqVe5Dh-WyVdV6bCSvRFNBGnUtyYqfiPYsc88uyf4CPvmM6q8RhFfbX1BEc-OwnRRePmUjT7EGFuTB6bcpW3yTvjQamkfBbGTrQow=&uniplatform=NZKPT&language=CHS>

**Abstract:**

Objective: To explore the effect of Xuesaitong injection on neurological function in patients with cerebral infarction. Method: Sixty patients with acute cerebral infarction admitted to our hospital were randomly divided into an experimental group and a control group. Both groups of patients received routine treatment for cerebral infarction, while the experimental group received Xuesaitong injection on this basis. Compare the plasma fibrinogen levels, neurological deficit score, and daily living ability score (Barthel score) between two groups of patients before and after treatment, and evaluate the clinical efficacy of the two groups of patients. Result: After treatment, the plasma fibrinogen level and neurological deficit score of the experimental group were significantly lower than those of the control group (P<0.05), while the treatment effective rate and Barthel score were significantly higher than those of the control group (P<0.05). Conclusion: Xuesaitong can significantly improve the neurological function and daily living ability of patients with cerebral infarction, enhance their clinical efficacy, and is worthy of clinical promotion and application,

**16. Yan, D., Li, Y., Zhang, L., Zhu, S., and Duan, J. (2020). The Effects of Xue-Sai-Tong Injection Combined with Intravenous Thrombolysis on Hemodynamics, Nerve Function, Serum Hcy, Nse and S-100β in Patients with Ischemic Stroke. Pract J Clin Med, 27–30.**

**URL:** <https://kns.cnki.net/kcms2/article/abstract?v=gPw7xOyBVLFYwx9R97XJQUgYouef0VVwSR-MpJhU6fJBorhsygQAYODj2YvsH9Q6WdI_Y-sNHNdslFG2RjunDn3dopdPrJ_FaZe5ZQMXxUe966ekiEmAX9jFAlLcw9gDfY0KilunYTab0c96Dx-Mpw==&uniplatform=NZKPT&language=CHS>

**Abstract:**

Objective: To explore the therapeutic effect of Xuesaitong injection combined with intravenous thrombolysis on patients with ischemic stroke. Method: 91 patients with ischemic stroke admitted to our hospital were randomly divided into a combination group (Xuesaitong injection combined with intravenous thrombolysis treatment) of 46 cases and a control group (intravenous thrombolysis treatment) of 45 cases using a random number table method. Compare the hemodynamics of two groups before and after treatment: the average blood flow velocity of the middle cerebral artery (MCA), anterior cerebral artery (ACA), and basilar artery (BA); Neurological function: National Institutes of Health Stroke Scale (NIHSS) score; Daily living ability: Barthel index (MBI) evaluation; Compare the levels of homocysteine (Hcy), neuron specific enolase (NSE), and serum protein (S-100B) between two groups before and after treatment. Result: After treatment, the average blood flow velocity of MCA, BA, and ACA in the combination group increased compared to the control group. The NIHSS score decreased compared to the control group, while the MBI index increased compared to the control group. The levels of Hcy, NSE, and S-1008 decreased compared to the control group (P<0.05). Conclusion: The combination of Xuesaitong injection and intravenous thrombolysis has a significant effect on the treatment of ischemic stroke, which can improve hemodynamics, protect neurological function, and enhance self-care ability.

**17. Zeng, L., Yang, S., Cui, W., Ban, Y., Gao, C., and Meng, X. (2023). The therapeutic effect of Xueshuantong injection combined with ateplase in the treatment of acute ischemic cerebrovascular disease. Liaoning J Tradit Chin Med, 1–9. doi: 10.13192/j.issn.1000-1719.2024.04.022**

**Abstract:**

Objective: To observe the clinical effect of Xueshuantong injection combined with ateplase in the treatment of acute ischemic cerebrovascular disease and its effect on serum tumor necrosis factor- α (TNF-α) and the impact of interleukin-1B (IL-1B). Method: A total of 90 patients with acute ischemic cerebrovascular disease were enrolled in the hospital from March 2018 to May 2021. They were randomly divided into an observation group and a control group, with 45 cases in each group, using a random number table method. The control group was treated with ateplase, while the observation group was treated with a combination of Xueshuantong injection. The course of treatment for both groups was 2 weeks. Compare the clinical efficacy of two groups, including neurological deficits (NIHSS) and ability to live (Barthel Index) score, hemorheology, and serum biochemical indicators (TNF-α), before and after 2 weeks of treatment, changes in IL-1B levels. Result: After 2 weeks of treatment, the total effective rate of the observation group was higher than that of the control group (P<0.05). Two sets of NIHSS scores, hemorheological indicators, and serum TNF-α, IL-1B levels decreased compared to before treatment, and the observation group was lower than the control group. BI scores increased compared to before treatment, and the observation group was higher than the control group (P<0.05). Conclusion: The combination of Xueshuantong Injection and Ateplase has a definite therapeutic effect on acute ischemic cerebrovascular disease, which can improve hemorheological indicators, inhibit inflammatory reactions, and reduce neurological damage.

**18. Zhang, J. (2023). Effects of Xuesaitong for Injection Combined with Alteplase Intravenous Thrombolysisand Aspirin in Treatment of Patients with Acute Cerebral Infarction. Med J Chinese People Health, 79–82. doi: 10.3969/j.issn.1672-0369.2023.14.024**

**Abstract:**

Objective: To observe the effect of injection of Xuesaitong combined with ateplase intravenous thrombolysis and aspirin in the treatment of patients with acute cerebral infarction. Method: A prospective study was conducted on 84 patients with acute cerebral infarction admitted to the hospital from October 2021 to October 2022. They were randomly divided into a study group and a control group, with 42 cases in each group, using a random number table method. The control group received intravenous thrombolysis with ateplase and routine antiplatelet therapy with aspirin, while the study group received treatment with Xuesaitong injection on the basis of the control group. The total effective rate of the two groups, the National Institutes of Health Stroke Scale (NIHSS) score, serum levels of nerve injury factors [central nervous system specific protein (S100B protein), neuron specific enolase (NSE)], coagulation function indicators [fibrinogen (FB), prothrombin time (PT), activated partial thromboplastin time (APTT), thrombin time (TT)], oxidative stress indicators [superoxide dismutase (SOD), glutathione] were compared. The levels of glutathione peroxidase (GSH px), total antioxidant capacity (T-AOC), malondialdehyde (MDA), and stroke specific quality of life scale (SS-00L) scores. Result: The total effective rate of the study group was 92.86%, which was higher than the control group's 73.81%, and the difference was statistically significant (P<0.05); After treatment, the NIHSS score and levels of S100B protein, NSE, FIB, and MDA in the study group were lower than those in the control group, while PT, APTT, and TT were longer than those in the control group. SOD, GSH px, T-AOC levels, and SS-00L were all higher than those in the control group, and the difference was statistically significant (P<0.05). Conclusion: The combination of Xuesaitong injection with ateplase intravenous thrombolysis and aspirin can improve the total effective rate and SS-00L score in patients with acute cerebral infarction, reduce NIHSS score, S100B protein level, NSE level, improve coagulation function indicators and oxidative stress indicators, and the effect is better than that of ateplase intravenous thrombolysis and aspirin treatment.

**19. Zhang, Y., Wang, Y., Song, Z., Li, R., and Wang, Y. (2022). The effect of butylphthalide combined with Xuesaitong injection on oxidative stress and cerebral hemodynamics in early acute cerebral infarction patients. Chinese J Difficult Diffic Cases 21, 247–251. doi: 10.3969/j.issn.1671-6450.2022.03.006**

**Abstract:**

Objective: To evaluate the clinical efficacy and safety of the combination of butylphthalide and Xuesaitong in the treatment of acute cerebral infarction patients, as well as its impact on serum oxidative stress indicators and cerebral hemodynamics. Method: 120 patients with first-time confirmed acute cerebral infarction admitted to the Neurology Department of the First Affiliated Hospital of Hebei North University from September 2018 to November 2020 were randomly divided into a control group and an observation group, with 60 cases in each group, using a random number table method. Both groups received routine treatments such as thrombolysis and antithrombotic therapy. The control group was given butylphthalide in addition to routine treatment, while the observation group was given Xuesaitong injection in addition to the control group. Both groups were treated for 7 days. Compare the National Institutes of Health Stroke Scale (NIHS) and Activities of Daily Living (ADL) scores, serum lipid levels (TC, TG, LDL-C, HDL-C), inflammatory indicators (hs CRP, IL6, TNF-a), oxidative stress indicators [superoxide dismutase (SOD), malondialdehyde (MDA), glutathione peroxidase (CSH Px)], and cerebral hemodynamic indicators [peak systolic blood flow velocity (Vs), diastolic blood flow velocity (Vd), mean blood flow velocity (Vm), and resistance index (RI)] before and after treatment in two groups to evaluate clinical efficacy and drug safety. Result: The total effective rate of the observation group was significantly higher than that of the control group (86.7% vs. 71.7%, P=0.043), and neither group experienced serious adverse drug reactions. After 7 days of treatment, the NIHSS score in the observation group significantly decreased compared to the control group, while the ADL score significantly increased. Observation group serum hS-CRP, IL6, TNF- α, the levels of MDA and GSH Px were significantly lower than those of the control group SOD was higher than the control group. The values of Vs, Vd, and Vm in the observation group were higher than those in the control group, while the RI values were lower than those in the control group. Conclusion: Early application of butylphthalide combined with Xuesaitong in the treatment of acute cerebral infarction patients can further improve their neurological function and ability to live, enhance clinical efficacy, have good safety, reduce inflammatory and oxidative stress reactions in the body, improve cerebral hemodynamics, and have good promotion and application value.

**20. Zhao F., Ma Y., and Wang W. (2024). Effect of Xueshuantong combined with alteplase in the treatment of acute cerebral infarction and its influence on cerebral hemodynamics. Clin Med Res Pract 9, 66-69+82. doi: 10.19347/j.cnki.2096-1413.202403017**

**Abstract:**

Objective: To analyze the effect of Xueshuantong combined with ateplase in the treatment of acute cerebral infarction and its impact on cerebral hemodynamics. Method: 200 patients with acute cerebral infarction admitted from April 2015 to October 2021 were selected as the study subjects, numbered, and randomly divided into a control group and an observation group, with 100 cases in each group. The control group received treatment with ateplase, while the observation group received combined treatment with Xueshuantong on the basis of the control group. Compare the therapeutic effects of two groups. Result: The total effective rate of treatment in the observation group was higher than that in the control group (P<0.05). After treatment, the whole blood high shear viscosity (HBV), whole blood low shear viscosity (LBV), hematocrit (HCT), and erythrocyte sedimentation rate (ESR) of the observation group were lower than those of the control group (P<0.05). After treatment, the average blood flow velocity (Vm) and systolic peak flow velocity (Vs) of the vertebral artery (VA) and bilateral middle cerebral artery (MCA) in the observation group were higher than those in the control group, and the National Institutes of Health Stroke Scale (NIHSS) score was lower than that in the control group (P<0.05). After treatment, the levels of S-100B, ET-1, SICAM-1, and NSE in the observation group were lower than those in the control group (P<0.05). There was no significant difference in the total incidence of adverse reactions between the two groups (P<0.05). Conclusion: The combination of Xueshuantong and Ateplase has a good therapeutic effect on acute cerebral infarction, which can improve the cerebral hemodynamics and hemorheological indicators of patients, enhance neurological function, and has ideal safety. It has important application value.
